# Human tau promotes Warburg effect–like glycolytic metabolism under acute hyperglycemia conditions

**DOI:** 10.1016/j.jbc.2025.108376

**Published:** 2025-03-05

**Authors:** Jinyi Yao, Keying Li, Zhenli Fu, Jingjing Zheng, Zicong Chen, Jiahao Xu, Guoqing Lai, Yaomin Huang, Jinsheng Huang, Guanying You, Shuangxue Han, Zhijun He, Qiong Liu, Nan Li

**Affiliations:** 1 Shenzhen Key Laboratory of Marine Biotechnology and Ecology, Brain Disease and Big Data Research Institute, College of Life Sciences and Oceanography, Shenzhen University, Shenzhen, China; 2 National R&D Center for Se-rich Agricultural Products Processing, Hubei Engineering Research Center for Deep Processing of Green Se-rich Agricultural Products, School of Modern Industry for Selenium Science and Engineering, Wuhan Polytechnic University, Wuhan, China; 3 Shenzhen-Hong Kong Institute of Brain Science-Shenzhen Fundamental Research Institutions, Shenzhen, China; 4 Instrumental Analysis Center of Shenzhen University, Shenzhen University, Shenzhen, China; 5 Shenzhen Bay Laboratory, Shenzhen, China

**Keywords:** tau, aerobic glycolysis, NAD^+^, oxidative phosphorylation, ROS, mTOR, Warburg effect

## Abstract

The neurofilaments formed by hyperphosphorylated tau is a hallmark of tauopathies. However, the biological functions of tau and the physiological significance of its phosphorylation are still not fully understood. By using human tau (441 a.a.) transgenic (hTau) mice, murine tau KO mice, and C57BL/6J (C57) mice, unexpectedly, we found that under acute hyperglycemia conditions, JNK but not previously reported GSK3β mediated tau phosphorylation. Moreover, Akt, the inhibitory kinase upstream of GSK3β, was activated in a tau-dependent manner. Furthermore, under acute high glucose conditions, the presence of human tau significantly augmented Akt activation but inhibited 4E-BP1 phosphorylation simultaneously, indicating that human tau is also involved in regulating the alternative activation of mTORC1/2. By comparing the hippocampal membrane-associated proteome, we found that human tau influenced the homeostasis of protein-membrane association under acute hyperglycemia conditions. Of note, with respect to C57 and Tau KO mice, the membrane association of oxidative phosphorylation–related proteins was impeded by human tau in the hippocampus. *In vitro* study consistently showed that aerobic glycolysis was promoted in the presence of human tau under high glucose conditions, which maintained the ratio of NAD^+^/NADH. On the other hand, human tau restricted the level of oxidative phosphorylation, modulated the activity of SDH, and reduced ROS production upon high glucose challenging. In summary, the current study revealed that human tau played an important role in regulating glycolytic metabolism under acute hyperglycemia conditions, which is similar with the Warburg effect, through influencing the homeostasis of protein-membrane association.

Tau was originally discovered as a microtubule-binding protein. Recently, many studies also showed that tau played an important role in insulin signaling pathway. For example, tau-deficient mice showed glucose intolerance and blunt response to insulin stimulation (1, 2, 3). The deficiency of tau also perturbed glucose-stimulated insulin secretion (4). Previous study demonstrated that tau inhibited the activity of phosphatase and tension homolog on chromosome 10. Therefore, loss of tau protein resulted in uncontrollable 3,4,5-phosphatidyl inositol triphosphate dephosphorylation and the attenuation of insulin signaling (5). The observations from mice model demonstrated that murine tau closely relates with insulin signaling pathway. However, the biological function may be strikingly different between murine tau and human tau, since the interactome of human tau showed that it also interacted with multitudinous mitochondrial proteins besides tubulin, including SDH, SGUCLG1, SUCLG2, SCL25A6, OXCT1, COX5B, VDAC2, and CYCS *et al.* (6) Moreover, the overexpression of human tau resulted in mitochondrial elongation and the reduction of mitofusion 2 ubiquitination (7). In flies, the expression of human tau also affected the expression of Drp1 and Marf (the homolog of human mitofusion 2) (8). These studies illustrated that human tau is also involved in the biogenesis of mitochondria.

In the field of tau-related neurodegenerative disease study, many efforts had been made to demonstrate how hyperphosphorylation of tau was triggered by stress, such as amyloid-beta, or hyperglycemia and the subsequent neurotoxicity induced by the seeding, propagation of aggregated tau. However, little is known about the physiological meaning of tau phosphorylation itself. The biological function of human tau is quite vague, mainly because of its flexible structure and the obscure numerous interacting proteins. In the current study, we determined the role of tau phosphorylation in brain glycolytic metabolisms by using acute hyperglycemia model and monitored the influences of human tau on insulin signaling pathway and the functions of mitochondria through comparing human tau (441 a. a.) transgenic mice (hTau), which do not express murine tau, with C57BL/6J (C57), tau knockout (Tau KO) at the very the early stage of hyperglycemia conditions.

To our surprise, under the acute hyperglycemia conditions (<14 days), it was shown that the phosphorylation of tau at multiple sites were mediated by JNK, but not GSK-3β as known in the prolonged (>40 days) hyperglycemia mice model induced by streptozotocin (STZ) treatment (9). On the other hand, the activation of Akt in brains, which is involved in mediating insulin signaling for glucose metabolism, was augmented by the presence of human tau under these conditions. *In vitro* study consistently showed that human tau strengthened high glucose–induced Akt activation in an mTORC2-dependent manner and facilitated aerobic glycolysis, thereby maintained NAD^+^/NADH ratio. Meanwhile, the oxidative phosphorylation level, as well as high glucose–induced reactive oxygen species (ROS) production, was limited by the presence of human tau. Collectively, here we identified that human tau is involved in regulating protein-membrane association, especially those related with insulin signaling, oxidative phosphorylation, fatty acid oxidation, ribosome, and proteasome. By this way, it promoted a Warburg effect–like glycolytic metabolism under acute hyperglycemia conditions.

## Results

### Acute hyperglycemia induced tau phosphorylation in brain

**Figure 1:**
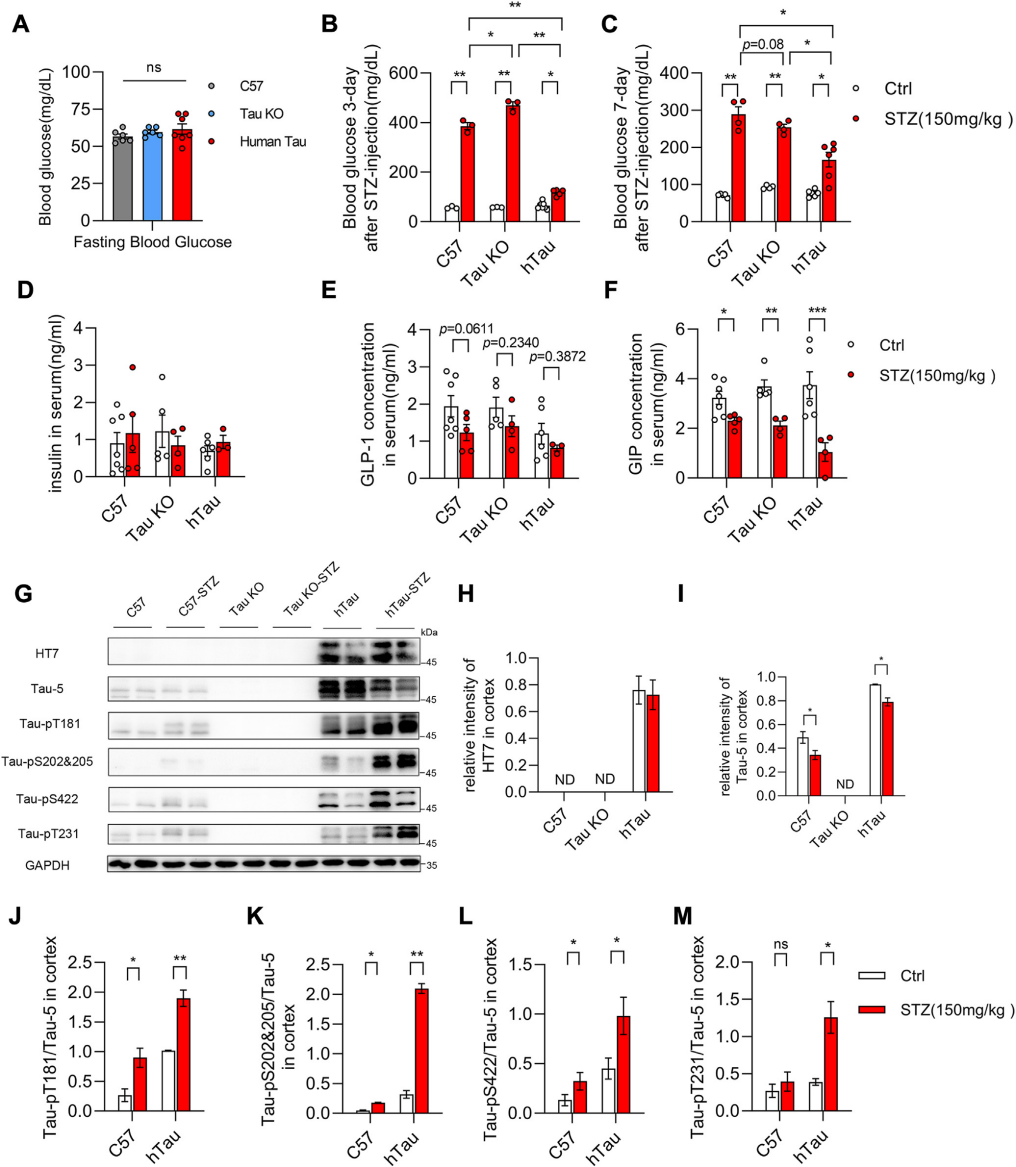
**STZ injection–induced acute hyperglycemia and tau phosphorylation.***A*, the levels of fasting-blood glucose in WT (n = 6), tau knockout (Tau KO, n = 6), and human tau transgenic (hTau, n = 7) mice. The results are shown as the mean ± s.e.m. by one-way ANOVA with Tukey's *post hoc* test. *B*, the levels of fasting-blood glucose in WT, Tau KO, and hTau mice 3 days after STZ (150 mg/kg) injection (WT, n = 3; Tau KO, n = 3; hTau, n = 5) compared with control groups (WT, n = 3; Tau KO, n = 3; hTau, n = 7). The results are shown as the mean ± s.e.m., ∗*p* < 0.05, ∗∗*p* < 0.01, by two-way ANOVA with Tukey's *post hoc* test. *C*, the levels of fasting-blood glucose in WT, Tau KO, and hTau mice 7 days after STZ (150 mg/kg) injection (WT, n = 4; Tau KO, n = 4; hTau, n = 6) compared with control groups (WT, n = 4; Tau KO, n = 4; hTau, n = 5). The results are shown as the mean ± s.e.m., ∗*p* < 0.05, ∗∗*p* < 0.01, by two-way ANOVA with Tukey's *post hoc* test. *D*–*F*, the levels of insulin (*D*), GLP-1 (*E*), and GIP (*F*) in nonfasting serum 7 days after STZ injection (WT, n = 5; Tau KO, n = 4; hTau, n = 3) compared with control groups (WT, n = 7; Tau KO, n = 5; hTau, n = 6). The results are shown as the mean ± s.e.m., ∗*p* < 0.05, ∗∗*p* < 0.01, ∗∗∗*p* < 0.001 by Mann Whitney two tailed nonparametric test. *G*, representative immunoblot of the lysate of cortex 7 days after STZ injection detected the protein level of human tau (HT7), total tau (tau-5). *H* and *I*, the protein level of human tau (*H*) was detected by HT7 (recognizes human tau specifically); the protein level of total tau (*I*) was detected by tau-5 (recognizes both human tau and murine tau). The protein level of GAPDH served as internal loading reference. The results are shown as the mean ± s.e.m. of tau/GAPDH, (WT STZ, n = 4; Tau KO STZ, n = 4; HTau STZ, n = 4; compared with equal number of controls, respectively), ∗*p* < 0.05, by Mann Whitney two tailed nonparametric test. *J*-*M*, the levels of phosphorylated tau/tau5 were detected by tau-pT181, tau-pS204&205, tau-pS422, tau-pT231 specific antibodies. The ratios of tau-pT181/tau-5 (*J*), tau-pS204&205/tau-5 (*K*), tau-pS422/tau-5 (*L*), tau-pT231/tau-5 (*M*) are shown as the mean ± s.e.m., (WT STZ, n = 4; Tau KO STZ, n = 4; HTau STZ, n = 4; compared with equal number of controls, respectively), ∗*p* < 0.05, by Mann Whitney two tailed nonparametric test.

Originally, the aim of this study was to monitor whether rapid tau phosphorylation induced by acute hyperglycemia would exhibit any neurotoxicity, especially through influencing mitochondria. Thus, human tau (441 a. a.) transgenic mice (hTau), tau knockout mice (Tau KO), and C57BL/6J mice (C57), which showed similar fasting blood glucose levels (Fig. 1*A*), were treated with intraperitoneal injection of STZ (150 mg/kg) to induce the hyperglycemia phenotype. This model has been widely used to study how insulin deficiency/resistance impacted on amyloid-beta production, tau phosphorylation, as well as the cognitive ability (9, 10). Previous reports demonstrated that STZ injection resulted in defects of insulin signaling, thus promoted tau phosphorylation in brain (4, 11). However, to our surprise, at the very early stage of hyperglycemia, that is 3 days (Figs. 1*B*), and 7 days (Fig. 1*C*) after STZ injection, the level of fasting blood glucose of hTau mice were much lower than that of Tau KO mice and C57 mice, though it was significantly upregulated comparing with non-STZ-injection group. To verify the effects of STZ treatment, we checked the levels of insulin (Fig. 1*D*), glucagon-like peptide-1 (GLP1) (Fig. 1*E*), and glucose-dependent insulinotropic peptide (GIP) (Fig. 1*F*) in the serum. Interestingly, in nonfasting serum, the levels of insulin and GLP1 were not affected, but the level of GIP was significantly reduced by STZ treatment for 7 days. We further examined the phosphorylation levels of tau in the cortex 7 days after STZ injection (Fig. 1*G*). It was shown by the immunoblot that human tau was only detected in the cortex lysates from hTau mice by HT7 antibody (Fig. 1*H*). Tau-5 antibody, which recognize both human tau and murine tau, reflected the lack of tau in the cortex lysates from Tau KO mice (Fig. 1*I*). Interestingly, Tau-5 also showed that STZ treatment reduced protein level of tau in the cortex, which was not revealed by HT7, indicating a subtle discrepancy of these two antibodies. While consistent with previous studies, the acute hyperglycemia induced increase of tau phosphorylation at multiple residues, including Thr181, Ser202/205, and Ser422 in the cortex of both C57 and hTau mice (Fig. 1, *J*–*L*), and the phosphorylation of Thr231 in the cortex of hTau mice (Fig. 1*M*).

To determine whether the effects of STZ treatment on the levels of fasting blood glucose and serum levels of insulin and GIP were repeatable, we employed 3xTg AD model mice, which also express 4R human tau (12) and their control WT mice. The level of fasting glucose of 3xTg AD mice were also significantly lower than that of WT mice 2 weeks after STZ injection (Fig. S1*A*), though they were all significantly upregulated by STZ treatment compared with their nontreated controls, respectively. Interestingly, the levels of insulin (Fig. S1*B*) and GIP (Fig. S1*C*) in nonfasting serum of 3xTg AD mice were significantly lower than that of WT mice. In addition, 2 weeks after STZ injection, the level of insulin in the serum of WT mice was decreased comparing with nontreated WT mice (Fig. S1*B*), which was not seen in C57 that treated by STZ for 7 days, suggesting that the downregulation of insulin by STZ treatment is behind of the decrease of GIP, while the level of GLP1 was decreased in both WT and 3x Tg AD mice compared with their control groups, respectively (Fig. S1*C*). We further examined the effect of hyperglycemia on tau phosphorylation in the cortex of WT mice and 3x Tg AD mice as well (Fig. S1*D*) and found that STZ treatment reduced the protein level of tau in the cortex of WT mice (Fig. S1*E*) but not in 3x Tg AD mice. We assume that may be because of the overexpression of human tau in these mice (Fig. S1*F*). Though WT mice did not express human tau (specifically recognized by tau-13 antibody), the murine tau could be recognized by the antibodies for phosphorylated tau. Consistently, STZ treatment for 2 weeks also resulted in increased tau phosphorylation at Thr181, Ser422, Ser404, and Ser396 in the cortex from 3x Tg AD mice and tau phosphorylation at Thr181 and Ser296 in the cortex of WT mice (Fig. S1, *G*–*J*).

### JNK mediated tau phosphorylation under acute hyperglycemia conditions

**Figure 2:**
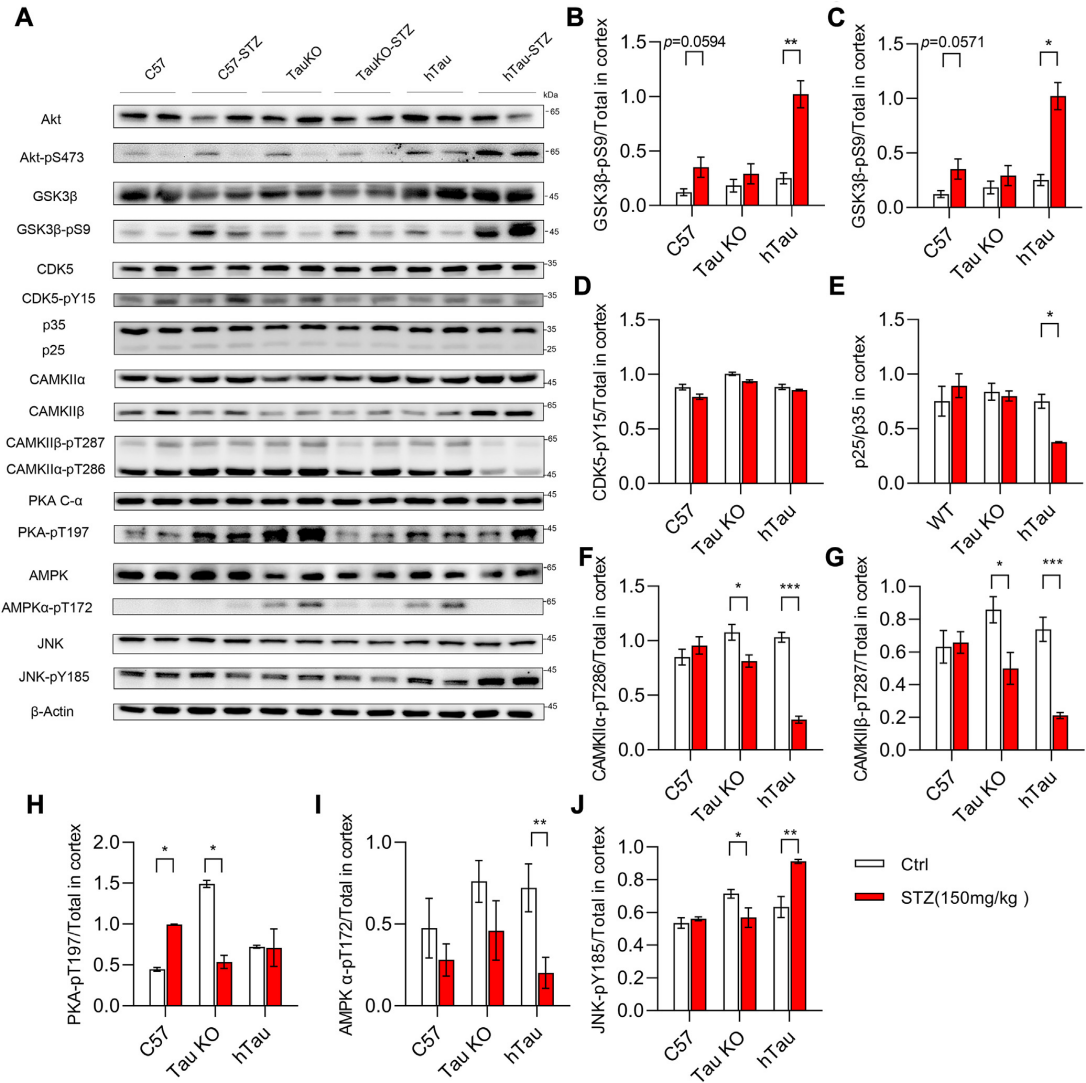
**STZ injection induced JNK activation in hTau mice.***A*, representative immunoblot of the cortex lysate 7 days after STZ injection from C57, Tau KO, and hTau mice compared with their control groups, respectively. The protein level of β-Actin served as internal loading reference. *B*–*J*, the ratio of Akt-pS437/Akt (*B*), GSK3β-pS9/tGSK3β (*C*), CDK5-pY15/CDK5 (*D*), p35/actin (*E*), CAMKIIα-pT286/CAMKIIα (*F*), CAMKIIβ-pT287/CAMKIIβ (*G*), PKA-pT197/PKA (*H*), AMPK-pT172/AMPK (*I*), and JNK-pY185/JNK (*J*) were analyzed and shown as the mean ± s.e.m., (WT, n = 4; Tau KO, n = 4; hTau, n = 4; compared with equal number controls, respectively), ∗*p* < 0.05, ∗∗*p* < 0.01, ∗∗∗*p* < 0.001 by Mann Whitney two tailed nonparametric test.

Previous studies demonstrated that STZ-induced long-term (>40 days) hyperglycemia caused tau phosphorylation through activating glycogen synthase kinase 3 beta (GSK3β), due to the deficiency of insulin and subsequent inactivation of Akt in brain (4, 11). To determine whether GSK3β was also activated under the acute hyperglycemia conditions, the phosphorylation levels of Akt and GSK3β were monitored by immunoblot (Fig. 2*A*). Unexpectedly, the phosphorylation of Akt at Ser473 was significantly increased (Fig. 2, *A* and *B*), and the inhibitory phosphorylation of GSK3β at Ser9 was also enhanced in the cortex of hTau mice 7 days after STZ injection (Fig. 2, *A* and *C*). These results, however, indicated that GSK3β may not account for tau phosphorylation shortly after STZ injection. Then, we further examined the phosphorylation levels of CDK5, PKA, CAMKII, AMPK, and JNK, as well as the protein levels of CDK5 activator, P35/P25, in the cortex by immunoblot. Interestingly, it was shown that the phosphorylation level of CDK5 (Fig. 2, *A* and *D*) and the protein level of p35 (Fig. 2, *A* and *E*) were not changed in the cortex 7 days after STZ injection. Moreover, the phosphorylation levels of CAMKIIα/β were inhibited in the cortex from Tau KO and hTau mice (Fig. 2, *F*, and *G*). Strikingly, the phosphorylation level of PKA was significantly higher in the cortex from Tau KO mice comparing with that of C57 and htau mice. Seven days after STZ injection, it was upregulated in C57 mice but decreased in Tau KO mice comparing with nontreated groups (Fig. 2, *A* and *H*), respectively, while the phosphorylation level of AMPK was significantly reduced in the cortex from hTau mice by STZ treatment compared with nontreated mice (Fig. 2, *A* and *I*). Among these kinases, only JNK was activated in the cortex of STZ-treated hTau mice compared with nontreated hTau mice (Fig. 2, *A* and *J*), indicating JNK may contribute to triggering tau phosphorylation under acute hyperglycemia conditions.

However, these results conflicted with the previous studies (4, 11), which showed GSK3β mediated tau phosphorylation after STZ treatment. We presumed that this may be because of the different time course between our experiment and others'. Therefore, we also compared the phosphorylation levels of Akt and GSK3β in the cortex of WT mice and 3xTg AD mice 2 weeks and 6 weeks after STZ injection with nontreated groups, respectively. As expected, the phosphorylation levels of Akt at Ser473 and GSK3β at Ser9 were decreased in the cortex from WT mice (Fig. S2, *A*–*F*) and 3xTg AD (Fig. S1, *G*–*L*) mice after long-term (6 w) STZ treatment, respectively. More importantly, we also found that the protein level of insulin-degrading enzyme was decreased upon STZ treatment in the cortex of either WT (Fig. S2, *A* and *E*) mice or 3x Tg AD mice (Fig. S2, *G* and *K*), and the phosphorylation of mammalian target of rapamycin (mTOR) was increased in the cortex of 3x Tg AD mice (Fig. S2, *G* and *J*) due to the less protein level of mTOR, but not in WT mice (Fig. S2, *A* and *D*). Astonishingly, the protein level of insulin was significantly increased in both of the long-term hyperglycemia groups (Fig. S2, *A*, *F*, *G*, and *L*), implying that the inactivation of Akt is caused by insulin resistant instead of insulin deficiency. Additionally, though the phosphorylation levels of JNK and p38 in the cortex of WT mice were not severely affected by STZ treatment (Fig. S3, *A*–*C*), we speculated that the transient activation of JNK by hyperglycemia may be sufficient for triggering tau phosphorylation at certain residues as seen in Figures 1*G* and S1*D*. However, in 3x Tg AD mice, 2 weeks after STZ injection, phosphorylation levels of JNK and p38 were increased, but 6 weeks after STZ injection, they were significantly reduced (Fig. S3, *F*–*H*) in the cortex compared with that from nontreated mice. Interestingly, the phosphorylation levels of CaMKII α/β were further reduced in both of the long-term STZ treatment groups (Fig. S3, *A*, *D*, *E*, *F*, *I*, and *J*), indicating that long-term hyperglycemia triggered impairment of neuroplasticity (13), which was not dependent on the existence of human tau.

### Tau phosphorylation induced by JNK promoted the alternative activation of mTORC2

**Figure 3:**
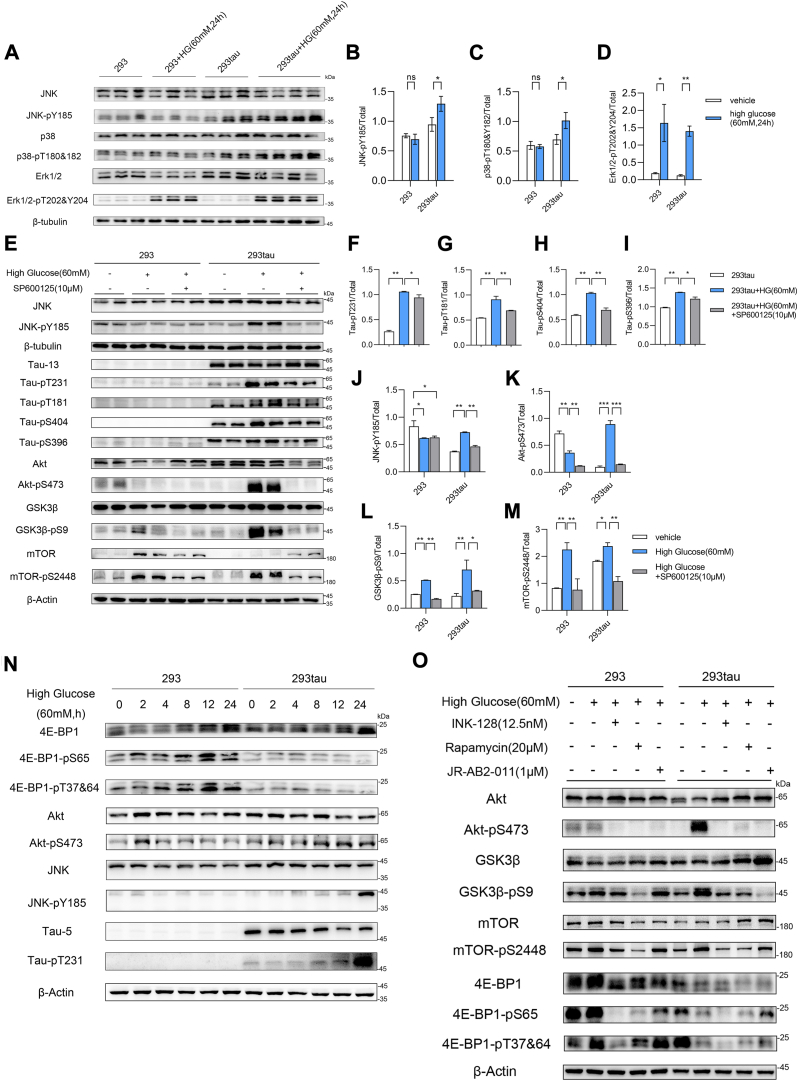
**Inhibition of JNK-arrested high glucose induced tau phosphorylation and tau-dependent Akt and mTOR activation.***A*, immunoblot analysis of MAPKs activation in normal HEK293 cells (293) and tau-expressing HEK293 cells (293tau) that were cultured in high glucose (60 mM) medium for 24 h compared with that cultured in normal medium. The protein level of β-Actin served as internal loading reference. *B*–*D*, the results of immunoblot are shown as the mean ± s.e.m. of duplicates (n = 3) for JNK-Py185/JNK (*B*), p38-pT180&182/p38 (*C*), and Erk1/2-pT202&204/Erk (*D*). ∗*p* < 0.05, ∗∗*p* < 0.01, ∗∗∗*p* < 0.001 by Mann Whitney two tailed nonparametric test. *E*, representative immunoblot analysis of the phosphorylation levels of JNK, tau, Akt, GSK3β, and mTOR and in normal HEK293 cells (293) and tau-expressing HEK293 cells (293tau) after 24 h of high glucose treatment (60 mM) combined with adding JNK inhibitor SP600125 (10 μM) in high glucose medium compared with that cultured in normal medium. The protein level of β-Actin served as internal loading reference. *F*–*M*, the results are shown as the mean ± s.e.m. of three independent experiments for tau-pT231/tau (*F*), tau-pT181/tau (*G*), tau-pS404/tau (*H*), tau-pS396/tau (*I*), JNK-pY185/JNK (*J*), Akt-pS473/Akt (*K*), GSK3β-pS9/GSK3β (*L*), and mTOR-pS2448/mTOR (*M*). ∗*p* < 0.05, ∗∗*p* < 0.01, ∗∗∗*p* < 0.001 by Mann Whitney two tailed nonparametric test. *B*–*D*, or by one-way ANOVA with Tukey's *post hoc* test (*F*–*M*). *N*, representative immunoblot of the normal HEK293 cells (293) and tau-expressing HEK293 cells (293tau) that were cultured with high glucose 0 to 24 h. The protein level of 4E-BP1, Akt, JNK, and tau, as well as the phosphorylation level of 4E-BP1 at S65 and at T37&64, Akt at S473, JNK at Y185, and tau at T231 were detected by individual antibody; β-Actin served as a loading control. The experiments were repeated for 3 times by using different batch of cells with similar results. *O*, representative immunoblot of the normal HEK293 cells (293) and tau-expressing HEK293 cells (293tau) that were treated with high glucose (60 mM) for 24 h, with or without mTORC1/2 inhibitor INK-128 (12.5 nM), mTORC1 inhibitor rapamycin (20 μM), mTORC2 inhibitor JR-AB2-011 (1 μM), respectively. The protein level of Akt, GSK3β, mTOR, 4E-BP1, the phosphorylation level of Akt at S473, GSK3β at S9, mTOR at S2446, 4E-BP1 at S65, and at T37&64 were detected; β-Actin served as a loading control. The experiments were repeated for three times by using different batch of cells with similar results.

To confirm whether JNK was responsible for tau phosphorylation under acute hyperglycemia conditions *in vitro*, we used a human tau (441 a. a.) overexpressing HEK293 cell line (239tau) and found that high glucose (60 mM) treatment for 24 h significantly induced JNK, p38, and Erk1/2 phosphorylation in these cells (Fig. 3, *A*–*D*), but only triggered Erk1/2 phosphorylation in normal HE293 cells (293) (Fig. 3, *A* and *D*). More importantly, high glucose also resulted in tau phosphorylation (Fig. 3, *E*–*J*), as well as tau dependent Akt, GSK3β, and mTOR phosphorylation (Fig. 3, *E*, *K*–*M*). Whereas, when JNK inhibitor SP600125 (10 mM) was added together with high glucose, the phosphorylation of tau was blocked efficiently along with the inactivation of JNK (Fig. 3, *E*–*J*). Meanwhile, JNK inhibitor also significantly arrested high glucose–induced Akt, GSK3β, and mTOR phosphorylation in 293tau cells (Fig. 3, *E*, *J*–*M*). These results indicated that JNK mediated tau phosphorylation under high glucose treatment, and the phosphorylation of tau is also essential for triggering mTOR and Akt activation under this stress. However, it is well known that mTOR participates in forming two complexes. Together with Raptor, mLST8, and others, they constitute mTORC1 which generates negative feedback to insulin signaling, which subsequently results in the inactivation of Akt but induces phosphorylation of 4E-BP-1, S6K. Together with Rictor, mLST8, and mSin1, and others, they form mTORC2 which facilitates phosphorylation of Akt on Ser473 residue (14). To demonstrate which complex is responsible for the activation of Akt in tau-expressing HEK293 cells, we compared the phosphorylation levels of 4E-BP1 and Akt in tau-expressing HEK293 cells and normal HEK293 cells that treated with high glucose for 0 to 24 h. The immunoblot results showed that high glucose induced 4E-BP1 phosphorylation only in normal HEK293 cell, whereas it led to Akt activation in both cell lines. However, in normal HEK293 cells, the phosphorylation of Akt was diminished soon, whereas it was prolonged along with the activation of JNK and the phosphorylation of tau (Fig. 3*N*). When INK-128, rapamycin, JR-AB2-011, the inhibitor of mTORC1/2, mTORC1, and mTORC2, respectively, were added together with high glucose, it was shown that INK-128 and JR-1B2-011 efficiently inhibited high glucose–induced Akt phosphorylation in tau-expressing HEK293 cells. Meanwhile, INK-128 and rapamycin significantly reduced high glucose–triggered phosphorylation of 4E-BP-1 in normal HEK293 cells (Fig. 3*O*). These results suggested that human tau promoted the alternative activation of mTORC2 under high glucose conditions, thereby prolonged high glucose–induced Akt activation. This is similar to the alterative activation of mTORC2 by posttranslational modification of Rictor and MLST8 (15, 16), of note, Rictor is also a tau interactor. However, the detail of underlying mechanisms needs to be further studied.

### Human tau promoted aerobic glycolysis under acute high glucose conditions

**Figure 4:**
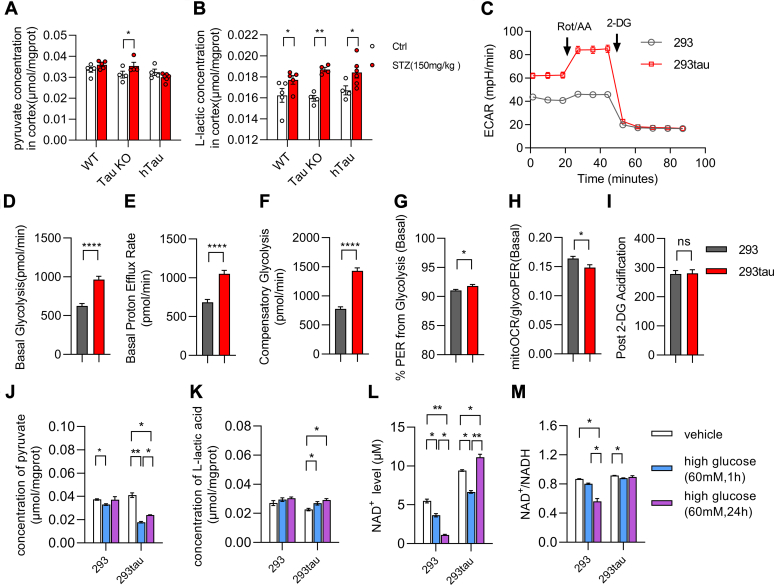
**Human tau promoted aerobic glycolysis.***A* and *B*, the concentration of pyruvate (*A*) and lactate (*B*) in the lysates of cortex 7 days after STZ injection (WT, n = 5; Tau KO, n = 4; hTau, n = 6) compared with control groups (WT, n = 5; Tau KO, n = 5; hTau, n = 6). The results are shown as the mean ± s.e.m., ∗*p* < 0.05, ∗∗*p* < 0.01, by Mann Whitney two tailed nonparametric test. *C*, representative bar graph of extracellular acidic rate (ECAR) trace of normal HEK293 cells (293) and tau-expressing HEK293 cells (293tau) measured by using the Seahorse apparatus. The experiments were repeated for three times by using different batch of cells with similar results. *D*–*K*, the levels of basal glycolysis (*D*), basal proton efflux rate (PER) (*E*), compensatory glycolysis (*F*), %PER from glycolysis (*G*), mitoOCR/glycoPER (*D*), and extracellular acidification rate after the hexokinase inhibitor 2-DG have been added (*I*) were compared between HEK293 cells (293) and tau-expressing HEK293 cells (293tau). The results are shown as the mean ± s.e.m. of the indicated time point, ∗*p* < 0.05, ∗∗*p* < 0.01, ∗∗∗∗*p* < 0.0001 by Mann Whitney two tailed nonparametric test. *J*–*M*, the concentration of pyruvate (*J*) and lactate (*K*) in the lysates of normal HEK293 cells and tau-expressing HEK293 cells that were treated with high glucose (60 mM) for 1 h or 24 h compared with that from normal medium cultured cells, respectively. The level of NAD^+^ (*L*) and the ratio of NAD^+^/NADH (*M*) in the lysates of normal HEK293 cells and tau-expressing HEK293 cells that were treated with high glucose (60 mM) for 1 h and 24 h compared with that from normal medium cultured cells, respectively. The results are shown as the mean ± s.e.m. of three independent experiments, ∗*p* < 0.05, ∗∗*p* < 0.01, one-way ANOVA with Tukey's *post hoc* test.

Under the acute hyperglycemia conditions induced by STZ injection, human tau enhanced Akt and mTOR activation as seen above. Due to the important roles of mTOR and Akt in mediating insulin signaling and facilitating glycolysis and tricarboxylic acid (TCA) cycle (17), we were curious whether tau exerts any influence on glycolysis under these conditions. Therefore, we examined the levels of pyruvate and lactate, the main products of glycolysis, in the cortex of mice brains. The level of pyruvate was significantly increased in the cortex from Tau KO mice upon STZ injection for 7 days. However, it was significantly lower in the cortex from hTau mice under the acute hyperglycemia conditions compared with those from C57 and Tau KO mice with the same treatment (Fig. 4*A*). The pyruvate produced by glycolysis will be further subjected to dehydrogenation to form lactate and replenish NAD^+^ or to form acetyl-CoA to participate in TCA cycle or biosynthesis, through the catalyzation of LDHA or PDK, respectively. Thus, we further compared the levels of lactate in the cortex. It was shown that in all three genotype mice, it was upregulated to a similar level 7 days after STZ injection (Fig. 4*B*).

It seems that the acidic circumstance could be restrained within a certain extend in the central nervous system. Thus, we decided to detect the impact of tau on the capability of glycolysis by performing extracellular acidification rate (ECAR) analysis *in vitro*. It was clearly shown that the basal glycolysis in human tau–expressing HEK293 cells (293tau) was significantly higher than that in normal HEK293 (293) (Fig. 4, *C* and *D*) as also reflected by the proton efflux rate (PER) (Fig. 4*E*). The level of compensatory glycolysis in tau-expressing HEK293 cells was also significantly greater than that in HEK293 cells, when oxidative phosphorylation was inhibited by rotenone/antimycin A (Rot/AA) (Fig. 4*F*). The %PER from glycolysis was also higher in tau-expressing HEK293 cells (Fig. 4*G*). However, the mitochondrial oxygen consumption rate/glycolysis PER was less in these cells compared with normal HEK293 cells, indicating a less oxygen consumption for glucose metabolism in tau-expressing cells (Fig. 4*H*). When glycolysis was inhibited by adding hexokinase inhibitor 2-DG, the ECAR were stopped in both cell lines (Fig. 4*I*), illustrating that the extracellular protons were indeed coming from aerobic glycolysis.

As mentioned above, pyruvate is subjected to dehydrogenation to form lactate in the process of aerobic glycolysis, which replenished NAD^+^ simultaneously. The *in vivo* data and ECAR results demonstrated that tau promoted Akt activation and aerobic glycolysis. Thus, we further detected the levels of pyruvate, lactate, and NAD^+^ in cells under high glucose (60 mM) stress. It was shown that the level of pyruvate in tau-expressing HEK293 cells was significantly reduced 1 h and 24 h after high glucose had been added comparing with those which were not treated with high glucose (Fig. 4*J*). Meanwhile, the level of lactate was increased along with the reduction of pyruvate (Fig. 4*K*). More importantly, 1 h after high glucose treatment, the level of NAD^+^ was decreased in both tau-expressing HEK293 and normal HEK293 cells compared with control group, respectively. However, 24 h after high glucose treatment, the level of NAD^+^ was increased significantly in tau-expressing HEK293 cells; in contrast, it went down continuously in normal HEK293 cells (Fig. 4*L*). The ratio of NAD^+^/NADH was also significantly reduced in normal HEK293 cells upon high glucose treatment for 24 h, but it was maintained by the presence of tau (Fig. 4*M*). These results demonstrated that human tau promoted aerobic glycolysis under high glucose conditions, which further transform pyruvate into lactate, so that the level of NAD^+^ was replenished efficiently.

### Human tau took part in regulating protein-membrane association

**Figure 5:**
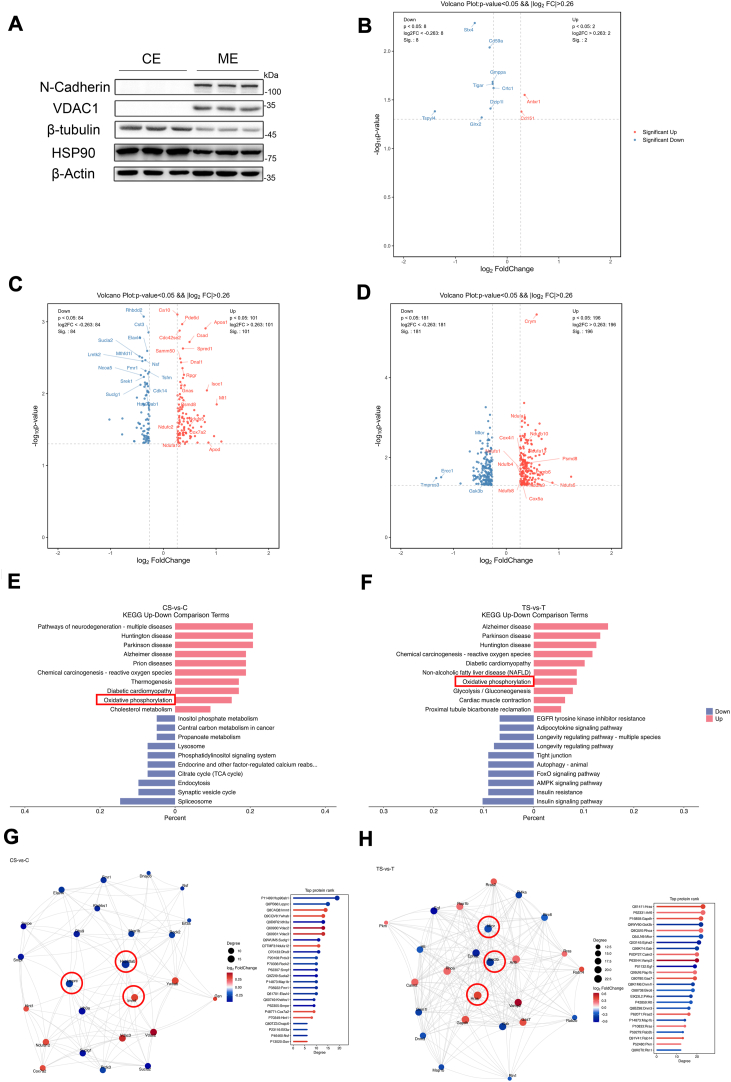
**Human tau maintained the homeostasis of protein membrane association in hippocampus under acute hyperglycemia conditions.***A*, membrane-associated proteins were isolated from the hippocampus and subjected to immunoblot for verification. The plasma membrane protein N-cadherin and the mitochondrial membrane protein VDAC1 were only detected in the membrane extract, but not in the cytoplasmic extract. However, the cytoskeleton protein, β-tubulin and chaperon protein HSP90 were more enriched in cytoplasmic extract, indicating the membrane-associated proteins were isolated successfully. *B*–*D*, volcano plots of the membrane-associated proteins (*p* < 0.05, log_2_ Fold Change>0.26) in the hippocampus from 4 months old male mice. Acute hyperglycemia induced by STZ injection for 7 days elevated two proteins but depressed eight proteins in association with membrane in hippocampus from hTau mice (n = 4), respectively, comparing with nontreated groups (n = 4) (*B*). STZ treatment for 7 days increased 101 proteins but reduced 84 proteins in association with membrane in the hippocampus of C57 mice (n = 4), respectively, comparing with nontreated C57 mice (n = 4) (*C*). Under the same conditions, 196 proteins were upregulated, but 181 proteins were downregulated in association with membrane in the hippocampus of Tau KO mice, respectively, comparing with nontreated Tau KO mice (*D*). *E*, KEGG analysis showed that with respect to control C57 mice, STZ treatment facilitated neurodegenerative disease and oxidative phosphorylation but restrained spliceosome-related proteins in association with membrane. *F*, KEGG analysis showed that with respect to control Tau KO mice, STZ treatment promoted neurodegenerative disease and oxidative phosphorylation but weakened insulin signaling pathway–related proteins in association with membrane. *G*, PPI network analysis revealed that HSP90ab1, LRPPRC, and IMMT are the top hub proteins in the network of discrepant membrane-associated between STZ-treated C57 mice and nontreated C57 mice. *H*, PPI network analysis uncovered Hras, GSK3β, and mTOR are hub note of differential membrane-associated protein network between STZ-treated tau KO mice and nontreated tau KO mice.

We presumed that tau-mediated aerobic glycolysis and activation of Akt attribute to the alternatively activation of mTORC2. It had been illustrated that the activation of mTOR is dependent on the association of mTOR with plasma membrane (18). In addition, tau was also found to interact with lipid membranes (19). Thus, for seeking the mechanism underlying tau-regulating kinase activity such as Akt, mTOR, and others, we further studied the discrepancy of membrane-associated proteome in hippocampus. The membrane-associated protein were isolated from hippocampus (Fig. 5*A*) and subjected to TMT-MS. Strikingly, it was shown that 7 days after STZ injection, in the hippocampus of hTau mice, only two proteins were upregulated and eight proteins were downregulated significantly in association with membrane comparing with nontreated hTau mice (Fig. 5*B*). Interestingly, one of the significantly downregulated proteins, Tigar, is related with metabolic switching from glycolysis to pentose phosphate pathway (20). However, under the same conditions, in the hippocampus of C57 mice, there were 101 and 84 membrane-associated proteins which were upregulated and downregulated, respectively, comparing with nontreated C57 (Fig. 5*C*). In addition, acute hyperglycemia resulted in 196 proteins more abundant on the membrane, but led to 181 proteins less combining with membrane in the hippocampus of Tau KO mice, comparing with nontreated Tau KO mice (Fig. 5*D*). KEGG analysis indicated that the membrane-associated proteins that were elevated by hyperglycemia in C57 and Tau KO mice are related with neurodegenerative disease, such as Alzheimer's disease (AD), Parkinson disease, Huntington disease (Fig. 5, *E* and *F*). This is consistent with the observations that these diseases are accompanied with dysregulation of glycose metabolism (21). Remarkably, the membrane association of oxidative phosphorylation–related proteins was also augmented by acute hyperglycemia in the hippocampus of C57 and Tau KO mice, but not in hTau mice (Fig. 5, *E* and *F*). Protein-protein interaction network analysis showed that the downregulation of Lrpprc, Hsp90a, and mTOR, GSK3β,which are all involved in regulating glucose metabolisms (22), played important role in the differential network of membrane-associated proteome under acute hyperglycemia conditions, in WT and tauKO mice, respectively (Fig. 5, *G* and *H*).

The proteome of membrane-associated proteins also showed that when comparing with C57 mice, in the hippocampus of Tau KO mice, 90 proteins were significantly more abundant in the membrane extract, including Zfyve19, Rpls, Psmds, and others; meanwhile, 90 proteins were significantly less associated with membrane, including Nnt, Mapt (tau), Trap1, GSK3β, and others (Fig. S4*A*). When comparing with C57 mice, 24 proteins were more abundant in the membrane extract, including Zfyve19, Mapt, TFAM, Krt13, and others; however, 39 proteins were less associated membrane, including Nnt, Ndufb1, Psmd5 and others, in the hippocampus of hTau mice (Fig. S4*B*). These results indicated that Nnt, Zfyve19 may not be affected by tau protein. Instead, we suspected that the human tau transgenic mice and tau KO mice may be derived from a strain of B6 mice, which do not express Nnt gene (23). When compared with Tau KO mice, on the other hand, the proteome showed that 209 proteins, including Mapt, RNF19a, Krts, Ercc1, TFAM, TRAP1, mTOR, and others, were more abundant on the membrane in the hippocampus of hTau mice. Interestingly, the membrane association of the JNK activator Map2k4 was also increased by human tau (The raw data of proteome are available at https://figshare.com/articles/dataset/human_tau_and_tau_knockout_mice_hippocampal_membrane_proteome_xlsx/27872493?file=50674347), which may explain why acute hyperglycemia triggered JNK phosphorylation in the brains of human tau–expressing mice. Whereas, 179 proteins, including Rpls, Psmds, Ndufb1, Ndufaf3, Cox5a1, Cox7a2, HSPs, and others, were less enriched on the membrane in the hippocampus of hTau mice (Fig. S4*C*). KEGG analysis demonstrated that deficiency of tau resulted in the elevation of ribosome-related protein but the decline of TCA cycle–related protein in the membrane extract of hippocampus comparing with C57 mice (Fig. S4*D*). It also showed that the proteins related to oxidative phosphorylation were less abundant on the membrane in hippocampus from the hTau mice comparing with both c57 and Tau KO mice (Fig. S4, *E* and *F*), indicating that human tau may interfere with oxidative phosphorylation. PPI network analysis illustrated that TRAP1, which is also known as a mitochondrial chaperone and involved in regulating respiratory capacity (24), was the top hub node in the differential membrane-associated proteome of hippocampus between Tau KO and C57 mice (Fig. S4*G*), while TFAM and mTOR played central roles in the differential membrane-associated proteome of hippocampus between hTau and C57 mice and between hTau and Tau KO mice (Fig. S4, *H* and *I*), respectively. Collectively, these results indicated that human tau possesses the ability of maintaining the homeostasis of protein-membrane association under acute hyperglycemia conditions, especially for the proteins that are involved in oxidative phosphorylation. It also directly or indirectly cooperated with the proteins that are involved in regulating glycolysis and mitochondria biogenesis, including TFAM, Tigar, TRAP1, mTOR, GSK3β, and others.

### Human tau restricted oxidative phosphorylation and ROS production

**Figure 6:**
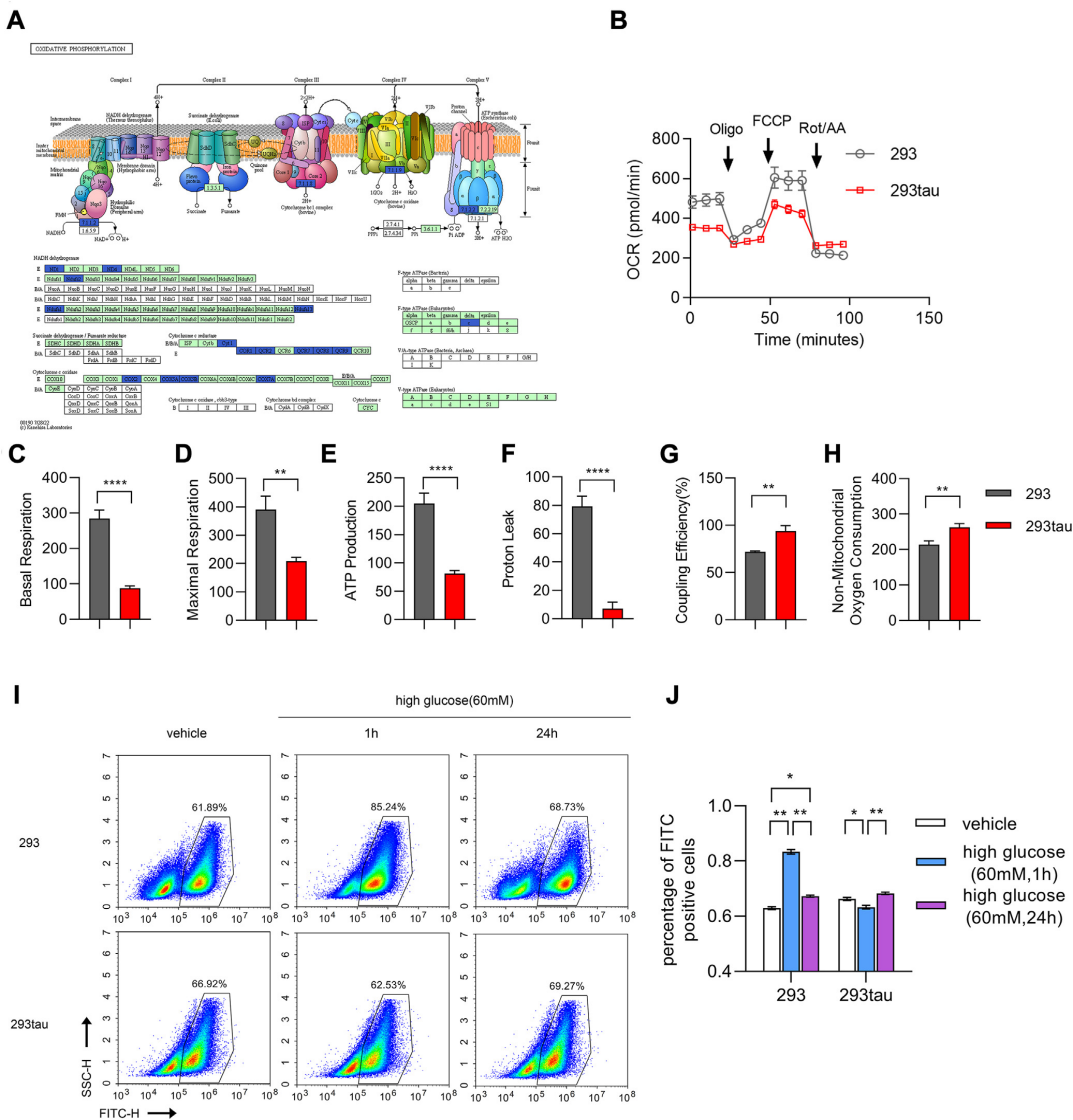
**Human tau restricted oxidative phosphorylation and inhibited acute high glucose–induced ROS production.***A*, the membrane-associated proteome showed that the components of NADH dehydrogenase, cytochrome c reductase, cytochrome c oxidase, and F-type ATPase were less abundant inassociation with membrane in hippocampus from hTau mice comparing with that from tau KO mice. *B*, representative graph of OCR from normal HEK293 cells (293) and tau-expressing HEK293 cells (293tau) measured by using the Seahorse apparatus. The experiments were repeated for 3 times by using different batch of cells with similar results. *C*–*H*, the oxygen consumption for basal respiration (*C*), maximal respiration (*D*), ATP production (*E*), nonmitochondrial (*F*), proton leak (*G*), as well as the coupling efficiency (*H*) were calculated and shown as the mean ± s.e.m. of the indicated time point. ∗*p* < 0.05, ∗∗*p* < 0.01, ∗∗∗∗*p* < 0.0001 Mann Whitney two tailed nonparametric test. *I*, the level of ROS in normal HEK293 and tau-expressing HEK293 cells that were treated with high glucose (60 mM) for 1 h and 24 h were detected with DCFH-DA and compared with normal medium-cultured cells, respectively. The percentage of fluorescence-positive cells were analyzed flow cytometric analysis. *J*, representative histogram and summary bar graphs showing frequencies of the cells stained with DCFH-DA (FITC) and shown as the mean ± s.e.m. of three duplications, ∗*p* < 0.05, ∗∗*p* < 0.01, by two-way ANOVA with Tukey's *post hoc* test. The experiments were repeated for three times by using different batch of cells with similar results.

As the membrane-associated proteome shown, many proteins that are involved in oxidative phosphorylation were less abundant in the membrane extract of hippocampus from hTau mice compared with that from Tau KO mice, including Mtnds, Ndufs, QCRs, Coxs, and others. (Fig. 6*A*). These results indicated that human tau (441 a.a.) may impede the functions of electron transport chain on the membrane of mitochondria. Therefore, we performed oxygen consumption rate (OCR) (Fig. 6*B*) to compare the level of oxidative phosphorylation in tau-expressing HEK293 (293tau) and normal HEK293 cell (293). As expected, the levels of basal respiration (Fig. 6*C*), maximal respiration (Fig. 6*D*), as well as the ATP production (Fig. 6*E*) of tau-expressing HEK293 cells were significantly lower than that of normal HEK293 cells as indicated by the less of O_2_ consumption. The proton leak, which switches the potential of proton to thermogenesis without ROS production, was restricted by tau as well (Fig. 6*F*). This is also consistent with the increased coupling efficiency in tau-expressing HEK293 cells compared with normal HEK293 cells (Fig. 6*G*). However, it was also shown that the nonmitochondrial oxygen consumption (Fig. 6*H*) was increased in tau-expressing HEK293 cells compared with normal HEK293 cells. We speculated that this is due to the upregulation of fatty acid oxidation as indicated by the membrane-association proteome which showed many proteins related to peroxisome (Fig. S5*A*), and fatty acid oxidation (Fig. S5*B*) were more abundant in membrane extract in the hippocampus from hTau mice comparing with that from Tau KO mice.

Since the oxidative phosphorylation is also a main source of ROS, which may induced the oxidation of lipids, DNA, and proteins when it is produced excessively, the level of ROS was further examined by using fluorescence probe 2′,7′-dichlorfluorescein-diacetate (DCFH-DA) under acute high glucose conditions. To our surprise, the results from flow cytometric analysis (Fig. 6*I*) showed that the frequency of DCFH-DA staining of tau-expressing HEK293 cells was higher than that of normal HEK293 cells. We assumed that this may also attribute to the augment of fatty acid oxidation in tau-expressing HEK293 cells. However, as expected, when challenged with high glucose for 1 h, the frequency of DCFH-DA staining was significantly increased in HEK293 cells; in contrast, it was restrained in tau-expressing HEK293 cells. We performed the fluorescence intensity detection by an Accuri C6 with the adherent-cultured HEK293 cells and human tau–expressing HEK293 cells (Fig. S6*B*) and got the similar results with flow cytometric analysis which indicated that human tau inhibited ROS production under acute high glucose conditions. Interestingly, upon challenging with high glucose for 24 h, the frequency of DCFH-DA staining in both of the cell lines all went back to normal level (Fig. 6*J*). These outcomes may be because of the transcription of antioxygenic proteins, such as heme oxygenase-1 as reported by previous studies (25, 26). Due to the proteome showed that the succinic dehydrogenase (SDH) A/B were less abundant in association with membrane in the hippocampus from hTau mice than that from Tau KO mice under acute hyperglycemia conditions (Fig. S6*A*). We further detected the activity of SDH, which is intimately related with not only electron transport chain but also TCA cycle (27). Surprisingly, the activity of SDH in tau-expressing HEK293 cells was significantly greater than that in normal HEK293 cell, which may interpret the higher coupling efficiency in the former cells as observed in OCR. On the contrary, 1 h after meeting with high glucose, the activity of SDH in human tau–expressing HEK293 cells was dramatically downregulated, and it was recovered 24 h after exposure to high glucose (Fig. S6*C*). These observations, therefore, demonstrated that human tau restricted the level of oxidative phosphorylation and it was also involved in regulating ROS production through mitochondrial respiration by influencing the activity of SDH.

## Discussion

It has been well established that human tau is involved in regulating glucose metabolism and mitochondrial functions. However, as a microtubule-binding protein, how does it contribute to these biological processes is not fully understood so far. Here, we showed that human tau promoted Warburg effect–like glycolytic metabolism under acute hyperglycemia conditions through modulating the homeostasis of protein-membrane association. Those proteins that were positively influenced by human tau for membrane association, including intermediate filament Kirts, heat shock proteins HSP90 and TRAP1, mitochondrial translational factor TFAM, and master signal transducer mTOR, showed close relations with glycolysis and mitochondrial homeostasis (28, 29). Those were negatively influenced by human tau comprised electro transport chain proteins, ribosome proteins Rpls (Fig. S7*A*), as well as proteasome proteins Psmds (Fig. S7*B*), indicating that except for impacting on oxidative phosphorylation, tau also participated in regulating protein synthesis and degradation. Of note, the dysfunction of ribosome and proteasome are also early events in AD (30, 31, 32). These results are consistent with tau interactome studies (33, 34), as well as the findings from analysis of tau evolution which indicated that human tau is involved in regulating protein-membrane association (35).

**Figure 7:**
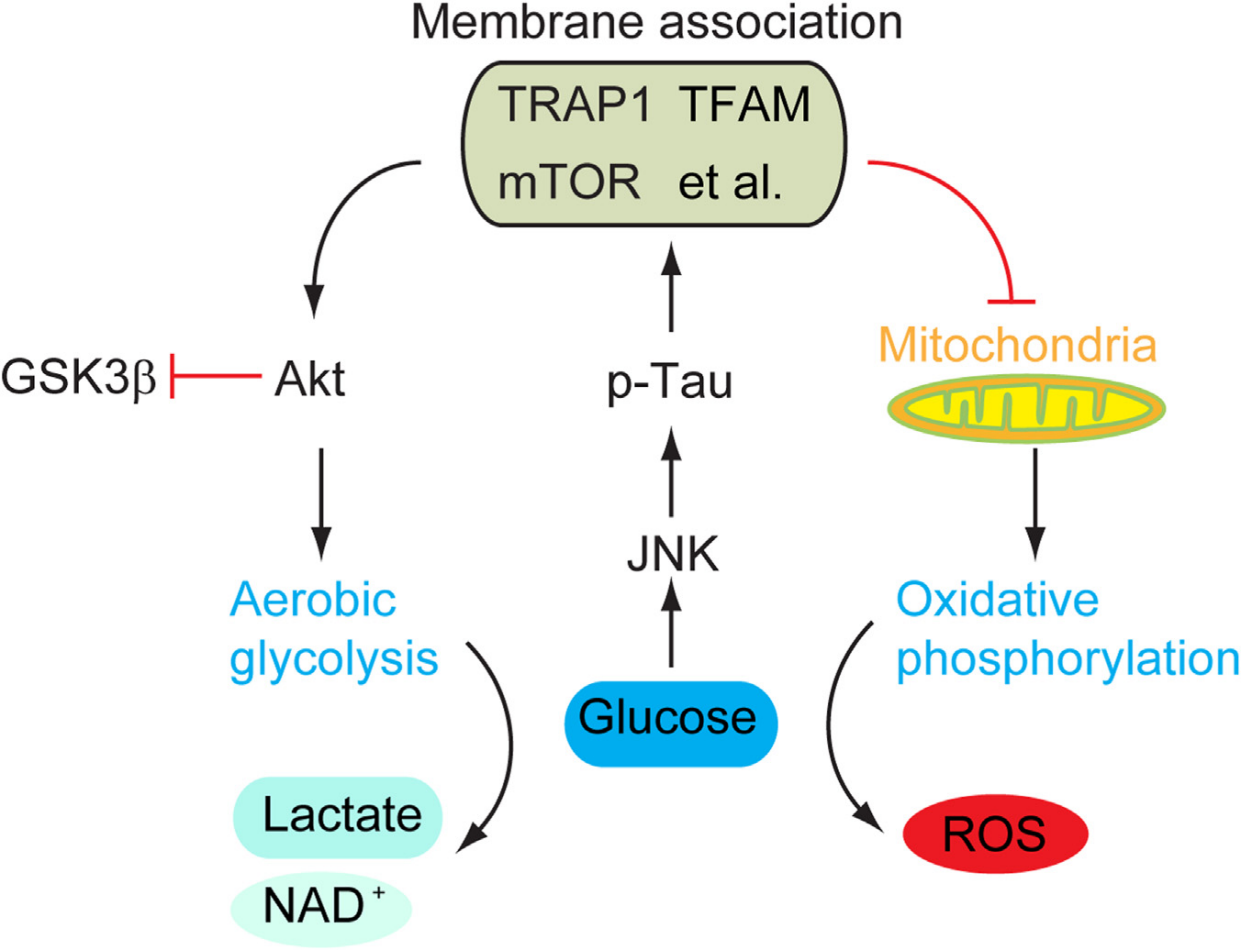
**Graphical abstract.** Under acute hyperglycemia conditions, high glucose stress activated MAPKs (JNK), which further induced tau phosphorylation. The phosphorylation of human tau maintained the membrane association of TRAP1, mTOR, TFAM, meanwhile restrained the membrane association of oxidative phosphorylation–related proteins. By this way, it facilitated aerobic glycolysis and restricted oxidative phosphorylation, which is similar with the Warburg effect.

Human brain consumed up to 60% of all blood glucose (36). The processes of glycolysis and fatty acid oxidation require the rapid replenish of NAD^+^. Meanwhile, ROS generated as a by-product of these metabolisms need to be severely restrained. In the current study, we observed that under acute hyperglycemia conditions, tau not only promoted Akt activation but also facilitated aerobic glycolysis, which further recovered the equilibrium of NAD^+^ level. Interestingly, it was shown that the activation Akt mediated by tau phosphorylation is dependent on the alternative activation of mTORC2. The membrane-proteome suggested that the membrane association of E3 ligase RNF19A, which has been demonstrated to be involved in regulating insulin signal, was significantly promoted by human tau. Thus, these results may further explain the insulin resistance observed in tau-deficient mice model. On the other hand, it restricted the level of oxidative phosphorylation and reduced ROS production through modulating protein-membrane association. Notably, it was shown that tau restricted ROS production very short after high glucose challenge (∼1 h) *in vitro*; however, it mediated Akt activation and NAD^+^ reproduction a little later (∼24 h) upon its phosphorylation. These observations indicated that the underlying mechanisms of human tau regulating aerobic glycolysis and oxidative phosphorylation probably through different signal pathway (Fig. 7). These finding are also consistent with previous observations that neuronal somata exhibited increased aerobic glycolysis and reduced OXPHOS in both basal and activated state (37) and are according with the glycolytic advantage seen in cancer cell attributing to Warburg effect (38), though the underlying molecular mechanism may be different. However, the aggregation of tau may result in its dysfunction in regulating protein-membrane association and subsequent energy metabolism deficit as seen in AD (39).

The reprogramming of glycolytic metabolism in AD has been revealed in recent years. For example, the induced neuron from AD patient-derived fibroblasts exhibited Warburg-like metabolic transformation through elevating PKM2 expression and its nuclear translocation (40). It has also been demonstrated that the lactate-dependent histone modification promoted metabolism reprograming in AD model, which could facilitate the activation of microglia (41). However, the driving forces of the metabolic reprogramming in neurodegenerative diseases are not clear. Based on the observations in the current study, we speculated that the phosphorylation of tau by JNK under physiological conditions plays important role in maintaining energy metabolism, particularly when blood glucose is elevated. However, the prolonged hyperphosphorylation of tau induced by other kinases and the aggregation of tau may give rise to the dysregulation of energy metabolism as well as the imbalance of protein synthesis and degradation in related neurodegenerative diseases. In this context, the prime option for the therapy of tauopathy should focus on preventing or inhibiting tau aggregation instead of eliminating tau, for example through antibodies. Because the level of of NAD^+^ and the balance of ROS are critical for the function of central nervous system, human tau is involved in regulating the equilibrium of these elusive small molecular.

## Experimental procedures

### Animal

Human tau transgenic mice (B6.Cg-Tg(MAPT) 8cPdav *Mapt*^*tm1(EGFP)klt*^*/*J, Strain: 005491) and murine tau KO (B6.129S4(Cg)-*Mapt*^*tm1(EGFP)klt*^*/*J, Strain: 029219) male mice were purchased from The Jackson Laboratory. C57 BL/6J mice were purchased from The Charles River. 3x Tg AD mice carrying the human mutations of APPswe, PS1M126V, and TauP301L (B6; 129-Psen1^tm1Mpm^ Tg (APPSwe, tauP301L)1Lfa/Mmjax. Strain:034830) were acquired from Jackson Laboratory. WT mice (B6129SF2/J, Strain: 101045) were used as controls for 3x Tg AD mice. All mice were housed in typical cages and lived in a humidity and temperature-normalized room with natural light-dark cycle which provided free water and food. All procedures were performed in compliance with guidelines approved by the Animal Ethical and Welfare Committee of Shenzhen University (Permit Number: IACUC-202300052).

For acute hyperglycemia induction, streptozotocin was diluted with citrate buffer (pH 4.2–4.5) to the final concentration (1%) before use. Four-month-old male mice of C57, Tau KO, and hTau mice were received intraperitoneal injection (150 mg/kg) and sacrificed 7 days after injection. Sex/age-matched mice of each genotype injected with the same volume of PBS served as control. Similarly, 3x Tg-AD and WT mice were received same injection and sacrificed two or six weeks thereafter. Female mice were not included in this model because they showed resistance to STZ-induced hyperglycemia.

### Chemicals and reagents

STZ were acquired from Sigma-Aldrich. Fetal bovine serum, Dulbecco's modified Eagle's medium (DMEM) high-glucose medium, and antibiotics (penicillin and streptomycin) were obtained from Gibco. ROS test kit was provided by Nanjing Jiancheng Bioengineering Institute. Pierce BCA protein assay kit was provided by Thermo Fisher Scientific. GIP, GLP-1 test kit were purchased from Jianglaibio. Pyruvate, lactate, and SDH assay kit were purchased from Elabscience. SP600125 (JNK inhibitor), INK-128 (mTORC1/2 inhibitor), rapamycin (mTORC1 inhibitor), and JR-AB2-011 (mTORC2 inhibitor) were procured from MedChemExpress.

### Cell culture

The HEK293 cell line that was stably transfected with the longest human tau (441 a. a.) cDNA (42) was provided by professor Jianzhi Wang from Tongji Medical College, Huazhong University of Science and Technology. Cells were cultured in DMEM supplemented with 10% fetal bovine serum and penicillin/streptomycin (Gibco) and maintained in a humidified atmosphere with 5% CO2 at 37 °C. Upon reaching 80% confluence, the cells were harvested and used for subsequent passage or high glucose with or without individual inhibitor treatment.

### Tissue/cell preparation and protein extraction

The mice were deeply anesthetized with isoflurane and euthanized. Brains were rapidly harvested, washed with normal saline, and then separated into cortex and hippocampus. The cortex from right hemispheres in each group were homogenized by sonification for 20 s in radio immunoprecipitation assay lysis solution containing 1 mM phenylmethylsulphonyl fluoride (Thermo Fisher Scientific) supplemented with phosphatase and proteinase inhibitors. Cells were washed twice with PBS and homogenized by sonification in radio immunoprecipitation assay premixed with a cocktail of inhibitors as above. Subsequently, the homogenates were centrifuged at 12,000 rpm for 30 min at 4 °C. The supernatants were collected and used for further experiments.

### Immunoblot analysis

Protein concentrations were determined by BCA protein assay kit. Tissue samples of equal amounts of proteins (20 μg) were loaded and separated on 8%, 10%, and 12% SDS-PAGE gel and then transferred to polyvinylidene fluoride membranes (Millipore) in the transfer buffer. After transferring, the polyvinylidene fluoride membranes were blocked with blocking buffer in tris-buffered saline containing 0.1% Tween 20 (TBST) for 30 min and then incubated with matched primary antibodies in TBST at 4 °C. After binding with the corresponding primary antibody, the membranes were washed with TBST for 30 min three times and incubated with the secondary antibody for another 2 h at room temperature. After three washes in TBST for 30 min, the protein bands were visualized using chemiluminescence with an ECL kit (Advansta), and the band intensities were analyzed using Image J software (NIH). β-Actin, β-tubulin, and GAPDH was detected as an internal loading control.

### Tissue membrane protein extraction

The hippocampi from 4-months-old male hTau (n = 4), Tau KO (n = 4), and C57 (n = 4) that were treated with STZ for 7 days and equal number controls of each group were used for membrane protein extraction in this study. Membrane and plasma extraction of brain tissues were obtained with the Mem-PER Plus kit (Thermo Fisher Scientific) according to the manufacturer's protocols. Briefly, the tissues were homogenized in permeabilization buffer following vortex in the cell wash solution. The sample were then incubated at 4 °C for 10 min and centrifuged at 16,000*g* for 15 min at 4 °C. The supernatant, which contained cytoplasmic proteins, was subsequently collected. The remaining precipitates were resuspended in a solubilization buffer and subsequently centrifuged at 16000g for an additional 15 min at 4 °C. The supernatant enriched with soluble membrane proteins and membrane-associated proteins was then collected. The final extractions were collected and used for further experiments.

### TMT-labeled quantitative proteomic

The Proteomic data analysis was performed by Shanghai Luming biological technology co, LTD. RP separation was performed on an 1100 HPLC System (Agilent) using an Agilent Zorbax Extend RP column (5 μm, 150 mm × 2.1 mm). Mobile phases A (2% acetonitrile in HPLC water) and B (90% acetonitrile in HPLC water) were used for RP gradient. The solvent gradient was set as follows: 0 ∼ 8 min, 98% A; 8 ∼ 8.01 min, 98% ∼ 95% A; 8.01 ∼ 48 min, 95% ∼ 75% A; 48 ∼ 60 min, 75 ∼ 60% A; 60 ∼ 60.01 min, 60 ∼ 10% A; 60.01 ∼ 70 min, 10% A; 70 ∼ 70.01 min, 10 ∼ 98% A; 70.01 ∼ 75 min, 98% A. Tryptic peptides were separated at a fluent flow rate of 300 μl/min and monitored at 210 nm. Samples were collected for 8 to 60 min, and eluent was collected in centrifugal tube 1 to 15 every minute in turn. Samples were recycled in this order until the end of gradient. The separated peptides were lyophilized for mass spectrometry.

All analyses were performed by a Q Exactive HF mass spectrometer (Thermo Fisher Scientific) equipped with a Nanospray Flex source (Thermo Fisher Scientific). Samples were prepared as demanded, thereafter were loaded and separated by a C18 column (50 cm × 75 μm) on an EASY-nLCTM 1200 system (Thermo Fisher Scientific). The flow rate was 300 nl/min and linear gradient was 75 min (0∼50 min, 2–28% B; 50 ∼ 60 min, 28–42% B; 60∼65 min, 42 ∼ 90%B; 65 ∼ 75 min, 90% B. mobile phase A = 0.1% FA in water and B = 0.1% FA in ACN). Full MS scans were acquired in the mass range of 350 to 1500 m/z with a mass resolution of 60,000 and the AGC target value was set at 3e6. All MS/MS pattern acquisitions were fragmented with higher-energy collisional dissociation with a collision energy of 32. MS/MS spectra were obtained with a resolution of 45,000 with an AGC target of 2e5 and a max injection time of 80 ms. The Q Exactive HF dynamic exclusion was set for 30.0 s and run under positive mode.

ProteomeDiscoverer (v.2.4.1.5) was used to search all of the raw data thoroughly against the Uniprot-*Mus musculus*-10090-2023.2.1. fasta database. Database search was performed with Trypsin digestion specificity. Alkylation on cysteine was considered as fixed modifications in the database searching. For protein quantification method, TMT was selected. A global false discovery rate was set to 0.01 and protein groups considered for quantification required at least one peptide. Annotation of all identified proteins was performed using GO (http://www.blast2go.com/b 2ghome; http://geneontology.org/) and KEGG pathway (http://www.genome.jp/kegg/). The differentially expressed proteins were further used for GO and KEGG enrichment analysis. PPI analysis was performed using the String (https://string-db.org).

### Determination of pyruvate, L-lactic acid levels

Brain tissues or cells were homogenized and centrifuged at 12,000 rpm for 20 min at 4 °C. Then, the supernatant was collected and the concentration of total protein was determined with a BCA assay kit. The levels of pyruvate and L-lactic acid were evaluated with pyruvate and L-lactic acid assay kits according to the manufacturer's protocols, respectively.

### Determination of NAD^+^ level

The levels of NAD+ in cells and tissues following glycolysis consumption were detected with a WST-8 kit (Beyotime). Briefly, cells or tissues were homogenized with NAD+/NADH extract from kit. Subsequently, the homogenates were centrifuged at 12,000*g* for 10 min at 4 °C. The supernatants were collected and split into two parts. One part of samples was heated to 60 °C to remove NAD^+^. Both parts and standards were incubated in ethanol dehydrogenase working solution in the dark for 20 min at 37 °C. Then the samples were incubated in electronically coupled reagents in the dark for 10 min at 37 °C. After incubation, the mean fluorescence intensity was detected at 450-nm using a spectra microplate reader (Thermo Fisher Scientific).

### OCR and ECAR analysis

OCR and ECAR were detected using seahorse XF24 analyzer (Seahorse bioscience). Briefly, HEK293 and HEK293-tau cells (2 × 10^4^ per well) were seeded in XF24 Cell Culture Microplates. Cells were covered with 475 μl assay medium (XF DMEM Medium, pH = 7.4), 10 mM glucose, 1 mM sodium pyruvate, and 1 mM L-glutamine. For OCR measurement, each port injection was processed with 1 μM oligomycin, 0.5 μM FCCP, 0.5 μM antimycin A, and rotenone. For ECAR measurement, glucose was elevated to 25 mM; each port injection was performed with 50 μM rotenone and 0.5 μM 2-DG.

### Flow cytometry standard

Human embryonic kidney (HEK-293/HEK-293tau) cells cultured in monolayer format within 6-well plates were exposed to high glucose DMEM (60 mM) at 37 °C for either one or 24 h. Postincubation, the cells were rinsed three times with PBS to eliminate residual media components. Thereafter, the culture medium was replaced with serum-free medium supplemented with DCFH-DA (Beyotime, Cat#S0033M) and incubated for an additional 30 min. This step facilitated the intracellular accumulation of the DCFH-DA probe, which is essential for the subsequent detection of ROS. To ensure that the probe was effectively internalized by the cells and to minimize extracellular background signal, the cells were collected and centrifuged at 1200 rpm for 5 min at 4 °C with PBS three times. After rinsing, the cells were resuspended in serum-free cell culture medium. Subsequently, cells were subjected to flow cytometry using the NovoCyte Advanteon Flow Cytometer (Agilent) equipped with NOVOExpress software for data acquisition and analysis.

### Determination of ROS level

The levels of ROS in cells following OXPHOS process were assessed with a fluorescent probe DCFH-DA kit. Briefly, the culture medium was removed and the cells were washed with PBS before the experiment. Then, the cells were cultured in serum-free medium containing 10 μM DCFH-DA dye in the dark for 20 min at 37 °C. After incubation, cells were washed twice with PBS to eliminate the extracellular DCFH-DA. The fluorescence intensity was detected at 488-nm excitation and 535-nm emission wavelengths by an Accuri C6 flow cytometry (Beckman Coulter).

### SDH activity assay

Cells were homogenized with reagents from SDH activity assay kit and then centrifuged at 600*g* for 5 min at 4 °C. Subsequently, the supernatants were collected and centrifuged at 15,000*g* for 10 min at 4 °C. Then the precipitates were sonicated for 5 min with new reagents from kit and centrifuged at 15,000*g* for 10 min. Finally, the supernatants were collected and the concentration of total protein was determined with a BCA assay kit. The SDH activity was detected at 600-nm by an Accuri C6 flow cytometry.

### Passivation of JNK and mTORC1/2 by inhibitor

The inhibitor was solubilized by DMSO (Yeasen) into an initial solution (the volume of DMSO was obtained by calculation from the MCE web site: https://www.medchemexpress.cn/molarity-calculator.htm). The supernatants of homogenates were collected after the cells were incubated for 24 h in high glucose medium (60 mM) containing the inhibitor. The working concentrations of each inhibitor were SP600125 (10 μM), INK-128 (12.5 μM), rapamycin (20 μM), and JR-AB2-011 (1 μM).

### Statistical analysis

All statistical analyses were conducted using GraphPad Prism 8.0 software (GraphPad). Results are presented as means ± s.e.m. Differences analyzed by utilizing Mann Whitney two tailed nonparametric test or one-way or two-way ANOVA with Tukey's *post hoc* test when appropriate. Significance was determined at *p* < 0.05.

## Data availability

The authors declare that the data supporting the findings of this study are available within the paper and the supplementary information files. The raw data of the proteome are deposited at https://figshare.com/articles/dataset/human_tau_and_tau_knockout_mice_ hippocampal_membrane_proteome_xlsx/27872493?file=50674347.

## Supporting information

This article contains supporting information.

## Conflict of interests

The authors declare that they have no conflicts of interests with the contents of this article.
