# Supplementary material for: Human tau promotes Warburg effect–like glycolytic metabolism under acute hyperglycemia conditions
Source: J Biol Chem. 2025 Mar 5;301(4):108376. doi: 10.1016/j.jbc.2025.108376 (PMC12018107; doi:10.1016/j.jbc.2025.108376)

**Supplementary Figures**


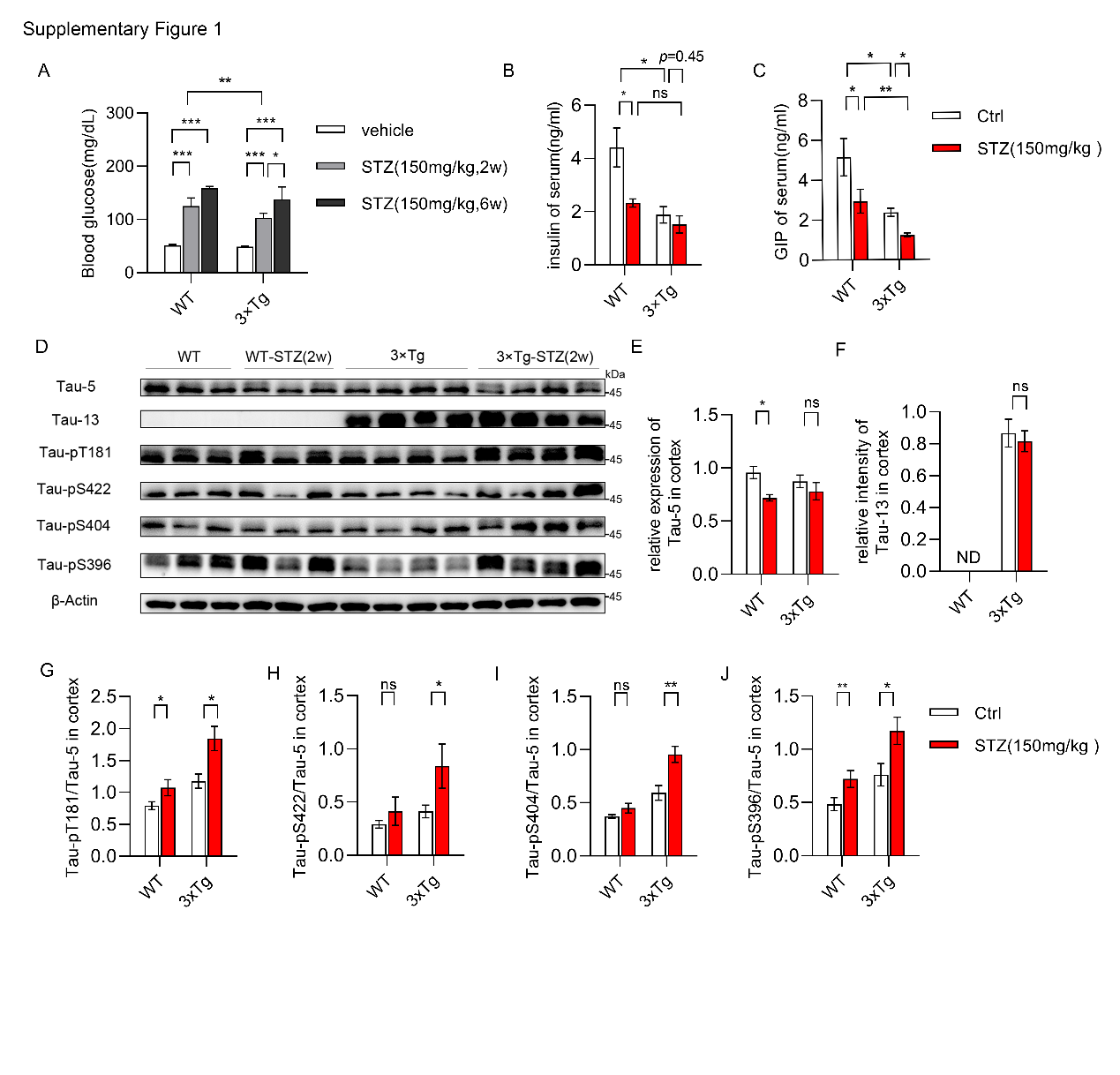


**Supplementary Figure 1 STZ injection induced tau phosphorylation in 3xTg AD mice**

**A,** The fasting blood glucose levels of WT and 3xTg AD model mice at 4 months of age were measured (WT, n=14. 3x Tg AD, n=18), and then these mice were subjected to STZ treatment, thereafter, the fasting blood glucose were measured at 2 w (WT, n=8. 3x Tg AD, n=11) as well as at 6 w (WT, n=6. 3x Tg AD, n=7) after STZ injection, respectively. The results are shown as the mean±s.e.m., **P*<0.05, ***P*<0.01, ****P*<0.001 by two-way ANOVA with Tukey’s post hoc test.

**B-C**, The levels of insulin (**B**) and GIP (**C**) in non-fasting serum of WT mice (n=3) and 3xTg AD model mice (n=6) that were injected with STZ (150 mg/kg) for 2 w, compared with that of WT mice (n=4) and 3xTg AD model mice (n=7) which were injected with PBS. The results are shown as the mean±s.e.m., **P*<0.05, ***P*<0.01, by two-way ANOVA with Tukey’s post hoc test.

**D,** Immunoblot and quantifications of the protein levels of total tau and the phosphorylation levels of tau. The protein level of β-Actin served as internal loading reference.

**E-J**, The ratio of total tau (tau-5)/ β-Actin (**E**), the ratio of human tau (tau-13) / β-Actin (**F**), the ratio of phosphorylated tau-pT181/total tau (tau-5) (**G**), the ratio of tau-pS422/total tau (tau-5) (**H**), the ratio of tau-pS404/total tau (tau-5) (**I**), the ratio of tau-pS396/total tau (tau-5) (**J**) in the lysates of cortex from WT mice injected with PBS (n=3), or STZ (n=3) for 2 w, as well as 3xTg AD model mice injected with PBS (n=4), or STZ (n=4) for 2 w. The results are shown as the mean±s.e.m., **P*<0.05, ***P*<0.01, by two-way ANOVA with Tukey’s post hoc test.


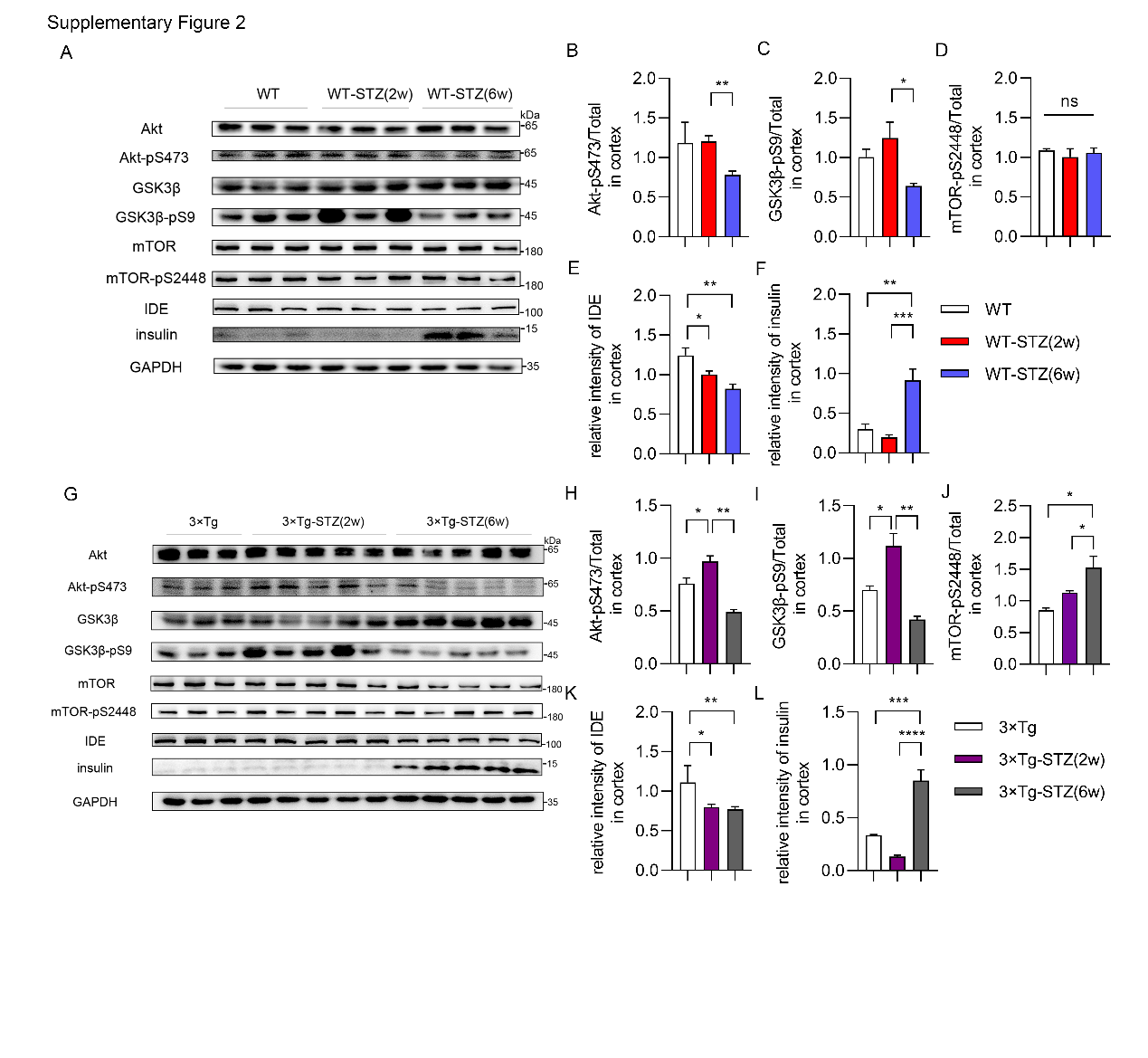


**Supplementary Figure 2 Prolonged hyperglycemia induced insulin resistance in brain**

**A,** WT mice injected with STZ for 2 w (n=3), and 6 w (n=3), respectively, the lysates of cortex were isolated and subjected to immunoblot analysis compared with that of WT mice (n=3) injected with PBS for 2 w. The protein level of GAPDH served as internal loading reference.

**B-F**, Quantifications of the blot were shown as the mean±s.e.m. of the ratios of Akt-pS473/Akt (**B**), GSK3β-pS9/GSK3β (**C**), mTOR-pS2448/mTOR (**D**), IDE/GAPDH (**E**), and insulin/GAPDH (**F**). **P*<0.05, ***P*<0.01, ****P*<0.001, by one-way ANOVA with Tukey’s post hoc test.

**G**, 3x Tg AD mice were injected with STZ for 2 w (n=5), and 6 w (n=5), respectively, the lysates of cortex were isolated and subjected for **i**mmunoblot analysis compared with that of 3x Tg AD mice (n=3) injected with PBS for 2 w (n=3). The protein level of GAPDH served as internal loading reference.

**H-L**, Quantifications the blot were shown as the mean±s.e.m. of the ratios of Akt-pS473/Akt (**H**), GSK3β-pS9/GSK3β (**I**), and mTOR-pS2448/mTOR (**J**), IDE/GAPDH (**K**), and insulin/GAPDH (**L**). **P*<0.05, ***P*<0.01, ****P*<0.001, *****P*<0.0001 by one-way ANOVA with Tukey’s post hoc test.


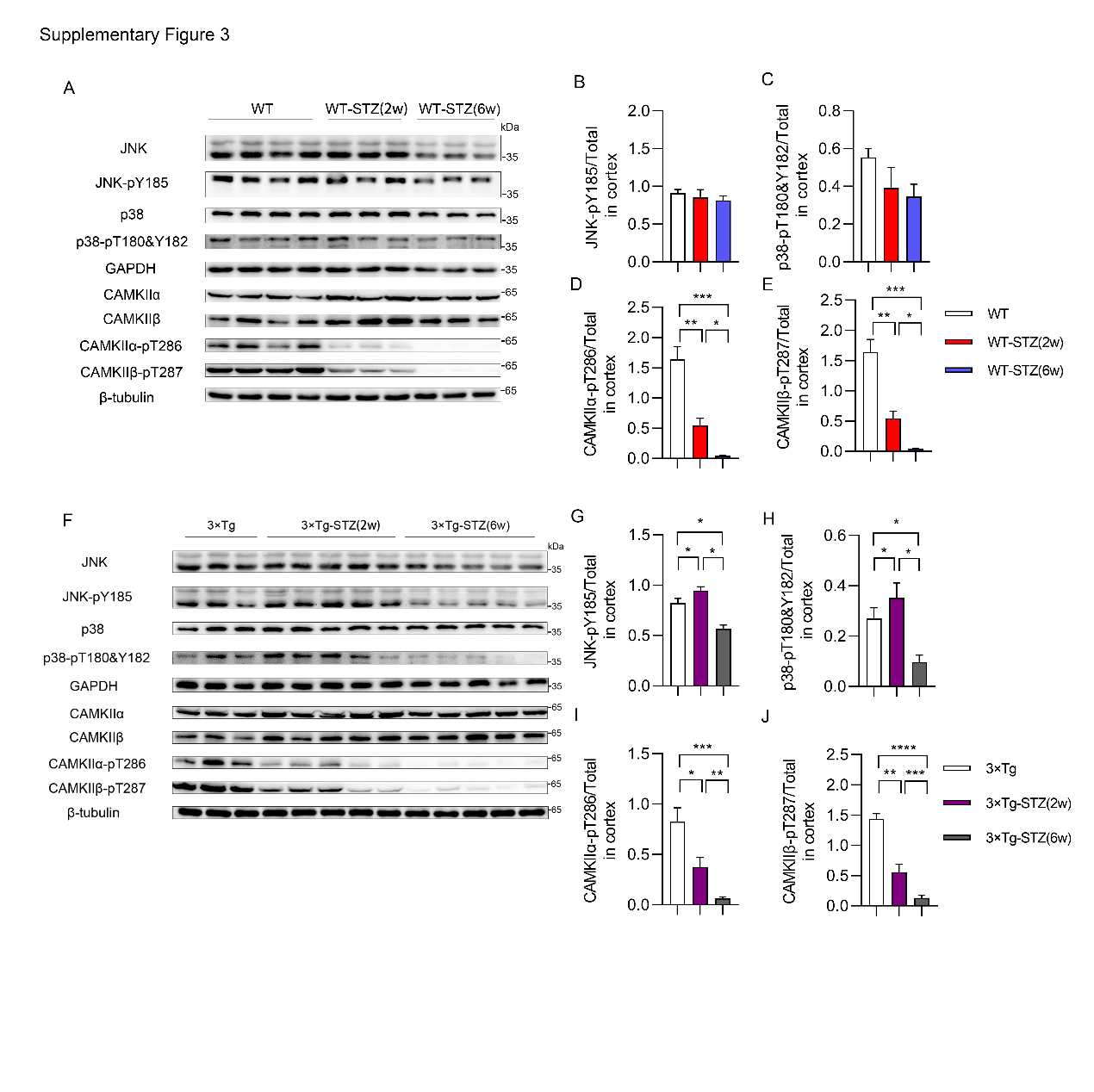


**Supplementary Figure 3 Prolonged hyperglycemia inhibited the phosphorylation of MAPKs and CaMKII**

**A,** WT mice injected with STZ for 2 w (n=3), or 6 w (n=3), respectively, the lysates of cortex were isolated and subjected for **i**mmunoblot analysis compared with that of WT mice (n=4) injected with PBS for 2 w. The protein level of β-tubulin served as internal loading reference.

**B-E**, Quantifications of the blot were shown as the mean±s.e.m. of the ratios of JNK-pY185/JNK (**B**), p38-pT180&182/p38 (**C**), CAMKIIα-pT286/CAMKIIα (**D**), and CAMKIIβ-pT287/CAMKIIβ (**E**). **P*<0.05, ***P*<0.01, ****P*<0.001, by one-way ANOVA with Tukey’s post hoc test.

**F**, 3x Tg AD mice were injected with STZ for 2 w (n=5), or 6 w (n=5), respectively, the lysates of cortex were isolated and subjected for **i**mmunoblot analysis compared with that of 3x Tg AD mice (n=3) injected with PBS for 2 w.

**G-J**, Quantifications the blot were shown as the mean±s.e.m. of the ratios of JNK-pY185/JNK (**G**), p38-pT180&182/p38 (**H**), CAMKIIα-pT286/CAMKIIα (**I**), and CAMKIIβ-pT287/CAMKIIβ (**J**). **P*<0.05, ***P*<0.01, ****P*<0.001, *****P*<0.0001 by one-way ANOVA with Tukey’s post hoc test.


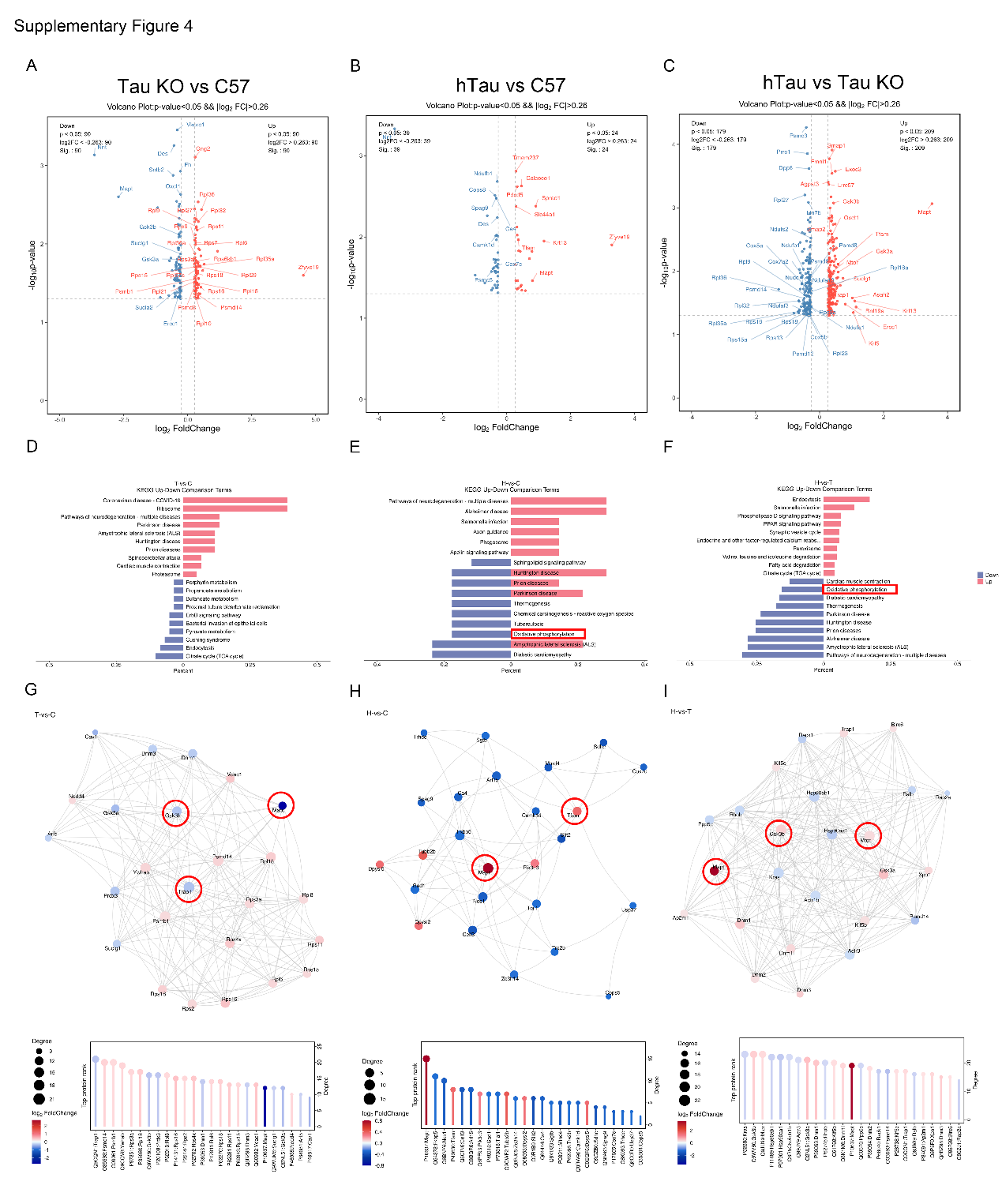


**Supplementary Figure 4 Tau altered the state of membrane-associated proteins in the hippocampus.**

**A-C,** Volcano plots of the membrane-associated proteins (*P*<0.05, log_2_ Fold Change>0.26) in the hippocampus from 4 months old male mice. By comparing with C57 mice (n=4), 90 proteins were significantly more abundant and 90 proteins were significantly less abundant in association with membrane in the hippocampus from tau KO mice (n=4) **(A)**. By comparing with C57 mice (n=4), 24 proteins were significantly more enriched and 39 proteins were significantly less enriched in association with membrane in the hippocampus from hTau mice (n=4) **(B)**. By comparing with Tau KO mice (n=4), 209 proteins were significantly more plentiful and 179 proteins were significantly less plentiful in association with membrane hippocampus from hTau mice (n=4) **(C)**.

**D-F,** KEGG analysis of the significantly up- and down-regulated membrane-associated protein. Compared with C57 mice, murine tau knockout upregulated ribosome, neurodegenerative diseases related proteins, but downregulated TCA cycle, pyruvate metabolism related proteins in associated with membrane **(D)**. Compared with C57 mice, human tau upregulated axon guidance, phagosome related proteins, but downregulated oxidative phosphorylation, thermogenesis related proteins in association with membrane **(E)**. Compared with murine tau knockout mice, human tau upregulated endocytosis, peroxisome, and fatty acid degradation related proteins, but downregulated oxidative phosphorylation, thermogenesis protein in association with membrane **(F)**.

**G-I,** Protein-protein interaction (PPI) network retrieved from the STING databases predicted the hub protein. In the differential membrane-associated protein network between tau KO and C57 mice, TRAP1, GSK3β, et al. played central roles **(G)**. In the differential membrane-associated protein network between human tau and C57 mice, MAPT, TFAM, et al. were the top rank hub **(H)**. In the differential membrane-associated protein network between hTau and tau KO mice, Kras, GSK3β, and mTOR were predicted as the most important hub proteins **(I)**.

**Supplementary Figure 5** (next page) **Human tau augmented the membrane-association of the peroxisome components and fatty acid degradation enzyme**

**A-B**, KEGG analysis membrane-associated proteome indicated that the components of peroxisome (**A**) and the enzymes that are involved in fatty acid degrading (**B**) were more abundant in association with membrane in the hippocampus from hTau mice than that from Tau KO mice. Significantly different proteins were indicated with red. The cells with green background indicated the proteins that did not show significant difference in the membrane extract of hippocampus between hTau mice and Tau KO mice. The cells with red background indicated the proteins that were significant more abundant in association with membrane in the hippocampus between hTau mice and Tau KO mice.

6.2.1.3: acyl-CoA synthetase long-chain family member 1,

1.3.8.7: acyl-Coenzyme A dehydrogenase,

1.3.8.8: acyl-Coenzyme A dehydrogenase,

2.3.1.16: acetyl-Coenzyme A acyltransferase 1A,

1.2.1.3: aldehyde dehydrogenase family 7, member A1.


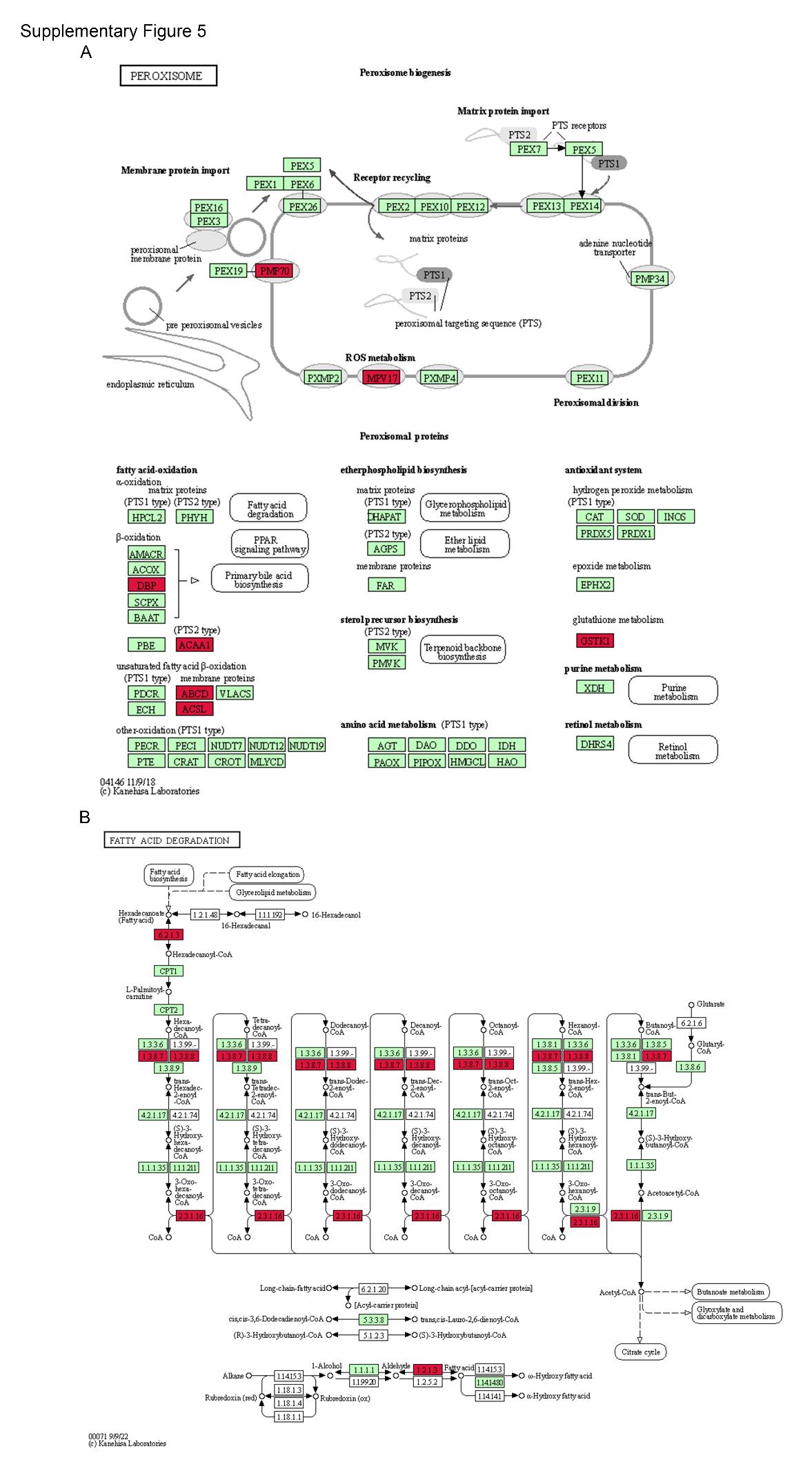


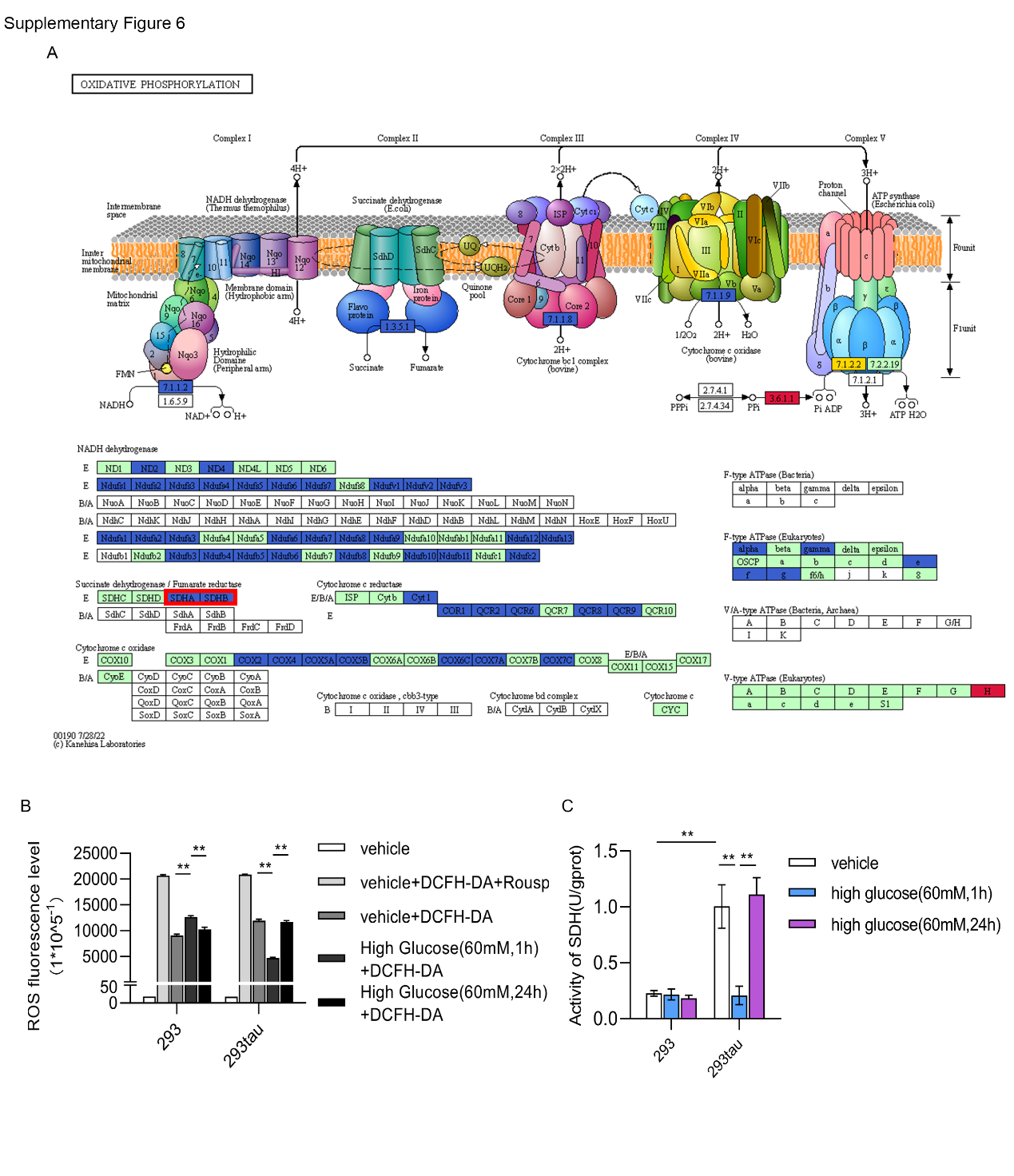


**Supplementary Figure 6 Tau knockout resulted in dysregulation of the oxidative phosphorylation related protein locating on membrane under acute hyperglycemia conditions**

**A,** KEGG analysis of membrane-associated proteome indicated that upon STZ treatment, more oxidative phosphorylation related proteins, including SDHA and SDHB (red frame) were less associated with membrane in the hippocampus from hTau mice than that from Tau KO mice. The cells with white background indicated the proteins that were not detected by TMT-MS. The cells with green background indicated the proteins that did not show significant difference in the membrane extract of hippocampus between hTau mice and Tau KO mice. The cells with blue background indicated the proteins that were significant less abundant in association with membrane in the hippocampus from hTau mice than that from Tau KO mice. The cells with red background indicated the proteins that were significant more abundant in association with membrane in the hippocampus of hTau mice compared with Tau KO mice.

**B,** The level of ROS in normal HEK293 and tau expressing HEK293 cells that were treated with high glucose (60 mM) for 1 h and 24 h were measured by fluorescent probe DCFH-DA and compared with normal medium cultured cells, respectively. Vehicle served as negative control, vehicle+DCFH-DA+Rousp served as positive control. The fluorescence was normalized to 10^-5^ cells and shown as the mean±s.e.m. of 3 duplications, **P*<0.05, ***P*<0.01, by two-way ANOVA with Tukey’s post hoc test. The experiments were repeated for 3 times by using different batch of cells with similar results.

**C**, The activity of SDH in the lysates of normal HEK293 (293) and human tau expressing HEK293 cells (293tau), which were treated with high glucose (60 mM) for 1 h or 24 h, were measured and compared with normal medium cultured cells, respectively. The results were normalized to per gram of protein and shown as the mean±s.e.m. of 3 duplications, ***P*<0.01, by two-way ANOVA with Tukey’s post hoc test. The experiments were repeated for 3 times by using different batch of cells with similar results.

**Supplementary Figure 7** (next page) **Human tau arrested the association of the of 26s proteasome and ribosome related proteins with membrane**

**A-B**, KEGG analysis of membrane-associated proteome indicated that the components of ribosome (**A**) and proteasome (**B**) were less abundant in association with membrane in the hippocampus from hTau mice than that from Tau KO mice. The cells with green background indicated the proteins that did not show significant difference in the membrane extract of hippocampus between hTau mice and Tau KO mice. The cells with blue background indicated the proteins that were significant less abundant in association with membrane in the hippocampus between hTau mice and Tau KO mice.


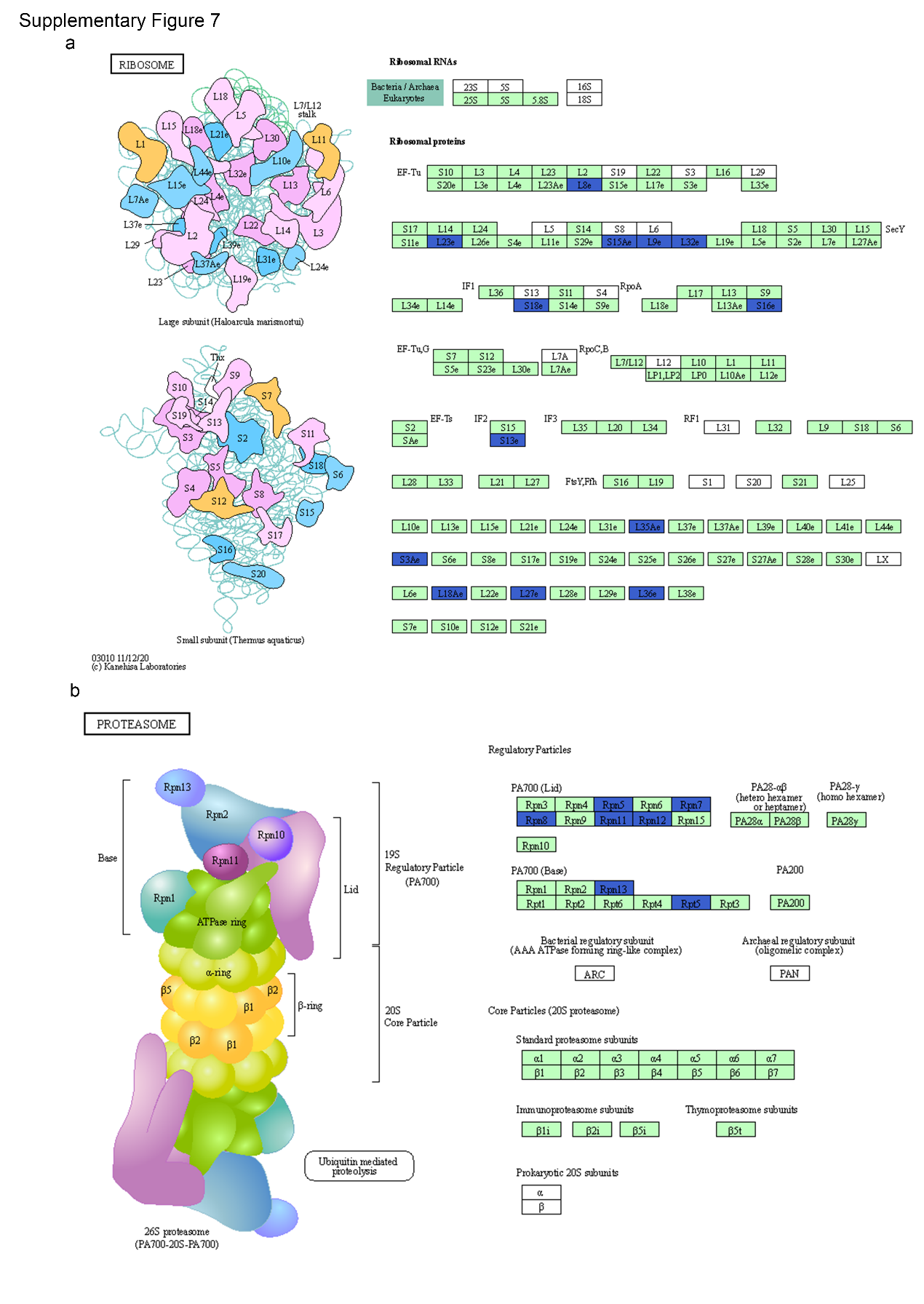

Supplement: Supplementary figures [file mmc1.docx]
